# Hexahydrocannabinol-induced rhabdomyolysis and acute kidney injury: a case report combining comprehensive toxicokinetic and metabolomic investigations

**DOI:** 10.1186/s42238-026-00435-7

**Published:** 2026-05-09

**Authors:** Pauline Thiebot, Romain Magny, Théo Willeman, Coralie Boudin, Margaux Brégère, Hélène Eysseric, Bruno Mégarbane, Nicolas Auzeil, Laurence Labat

**Affiliations:** 1 Laboratoire de Toxicologie Biologique, Fédération de Toxicologie, Hôpital Lariboisière AP-HP, Paris, France; 2 INSERM UMRS-1144, Université Paris Cité, Paris, France; 3 Laboratoire de Pharmacologie, Pharmacogénétique Et Toxicologie, CHU Grenoble Alpes, Grenoble, France; 4 Laboratoire de Médecine Légale, Université Grenoble Alpes, Grenoble, France; 5 Réanimation Médicale Et Toxicologique, Hôpital Lariboisière AP-HP, Fédération de Toxicologie, Paris, France; 6 CiTCoM, CNRS, Université Paris Cité, Paris, France

**Keywords:** Case report, Hexahydrocannabinol, Semi-synthetic cannabinoid, Untargeted metabolomic, Molecular network, Endometabolome, Seizure, Rhabdomyolysis, Acute kidney injury, UHPLC-ESI-HRMS

## Abstract

**Background:**

Following the recent amendments in international legislations on cannabis, semi-synthetic cannabinoid derivatives have become increasingly available worldwide. These new psychoactive substances are obtained through chemical modifications of cannabidiol (CBD), a widely available legal compound, or through chemical synthesis from fitting precursors. Compared to Δ^9^-tetrahydrocannabinol (Δ^9^-THC), the primary psychoactive constituent of *Cannabis sativa*, they usually display more potent effects and threatening toxicities, raising safety concerns thus requiring strengthened vigilance and monitoring. In the present study, we investigated a reported case of hexahydrocannabinol (HHC) poisoning, using a comprehensive toxicokinetic and metabolomic approach.

**Case presentation:**

A 35-year-old chronic tobacco and HHC male user experienced after smoking products a tonic–clonic seizure followed by a coma. His clinical course was marked by severe rhabdomyolysis, leading to acute kidney injury. The patient recovered without dialysis and was discharged after an 15-day stay in the hospital, including 11 days in the ICU. Toxicological monitoring was conducted through serial plasma sampling throughout his hospitalization.

**Methods:**

Xenometabolome and endometabolome were determined using untargeted metabolomic analysis combined to molecular networking. The assay was based on ultra-high performance liquid chromatography hyphenated to high resolution mass spectrometry data acquisition.

**Results:**

Thirty-five HHC metabolites including hydroxylated, carboxylated and glucuronoconjugated forms were identified in plasma and urine, similarly to Δ^9^-THC metabolites. A correlation of HHC concentration with phenylalanine level and of creatine kinase and creatinine concentrations with carnitine and dicarboxylic acids were observed.

**Conclusions:**

This case report of HHC intoxication provides a detailed clinical description. The toxicological exploration of biological matrices enabled the identification of HHC metabolites. Based on metabolomic analysis, physiological perturbations of biochemical pathways, such as phenylalanine metabolism, carnitine pathway, β-oxidation and ω-oxidation were highlighted. They corroborate recent findings from in vitro and in vivo studies, and provide new insights into the consequences of cannabinoid intoxication.

**Supplementary Information:**

The online version contains supplementary material available at 10.1186/s42238-026-00435-7.

## Highlights

• Hexahydrocannabinol exposure has been associated with tonic-clonic seizures complicated by rhabdomyolysis and acute kidney injury.

• Untargeted metabolomic analysis combined to molecular networking is an effective tool for exhaustive xenometabolome and endometabolome characterization.

• Comprehensive description of hexahydrocannabinol metabolism confirm a metabolism similar to that of Δ^9^-tetrahydrocannabinol, with identification of 35 hydroxylated, dihydroxylated and carboxylated metabolites and some of their glucuronides.

• Hexahydrocannabinol toxicity combines alterations in phenylalanine and carnitine metabolisms, beta-oxidation and omega-oxidation.

**Supplementary Information:**

The online version contains supplementary material available at 10.1186/s42238-026-00435-7.

## Introduction

Δ^9^-tetrahydrocannabinol (Δ^9^-THC), the primary psychoactive compound in *Cannabis sativa*, is the most widely consumed illicit drug, with 228 million users worldwide in 2022 (United Nations Office on Drugs and Crime 2024). Since the early 2020s, evolving cannabis legislation has increased its accessibility and sparked growing interest in its use, while promoting the availability of other cannabinoids such as cannabidiol (CBD). To appeal to consumers and comply with regulations, synthetic cannabinoids (SCs) and semi-synthetic cannabinoids (SSCs) have been developed and marketed. They are classified as new psychoactive substances (NPS). SCs exhibit a higher affinity for CB1 and CB2 receptors and are often more potent than Δ^9^-THC (Persson et al. 2024; Russo et al. 2023). They trigger a wide range of serious adverse effects including neurological (e.g., drowsiness, dizziness, agitation, hallucinations, confusion and psychosis), cardiovascular (e.g., tachycardia, chest pain and acute coronary syndrome), gastrointestinal (e.g., nausea and vomiting), and other organ dysfunctions such as respiratory depression, liver injury, rhabdomyolysis and acute kidney injury (AKI) (Kourouni et al. 2020). Nausea/emesis and abdominal/flank pain represent the most frequently observed side effects, followed by the neurological then urological symptoms. In most NPS poisonings—whether involving (S)SCs or other drugs—management is primarily supportive. Identifying the exact drug responsible for toxicity is based on toxicological analyses aiming to screen the parent substance or metabolites in the patient’s biological fluids including urine, saliva or blood. However it is rarely obtained in a compatible timeframe (Kourouni et al. 2020; Amanollahi et al. 2023). This is particularly true for SSCs, which identification in biological matrices remains challenging. SSCs are rapidly metabolized by phase I and II enzymes, leading to multiple metabolites that quickly become the sole indicators of exposure (D’Errico et al. 1936; Znaleziona et al. 2015). Moreover, new SSCs are continuously introduced on the market to evade governmental sale restrictions. An appropriate analytical strategy such as an untargeted metabolomic approach using ultra-high performance liquid chromatography with electrospray ionization source and high-resolution mass spectrometry (UHPLC-ESI^±^-HRMS) is thus suitable to address the extensive structural diversity arising from the multitude of SSCs and metabolites, namely the xenometabolome. Of note, in the absence of any information regarding the drug consumed, metabolomic analysis should benefit from molecular networks and databases of appropriate chemical structures to enable rapid and reliable drug identification (Magny et al. 2023). Such an approach additionally allows characterizing the endometabolome and investigating metabolic pathways as potential biomarkers of exposure/monitoring when managing the patient (Magny et al. 2022).

**Fig. 1:**
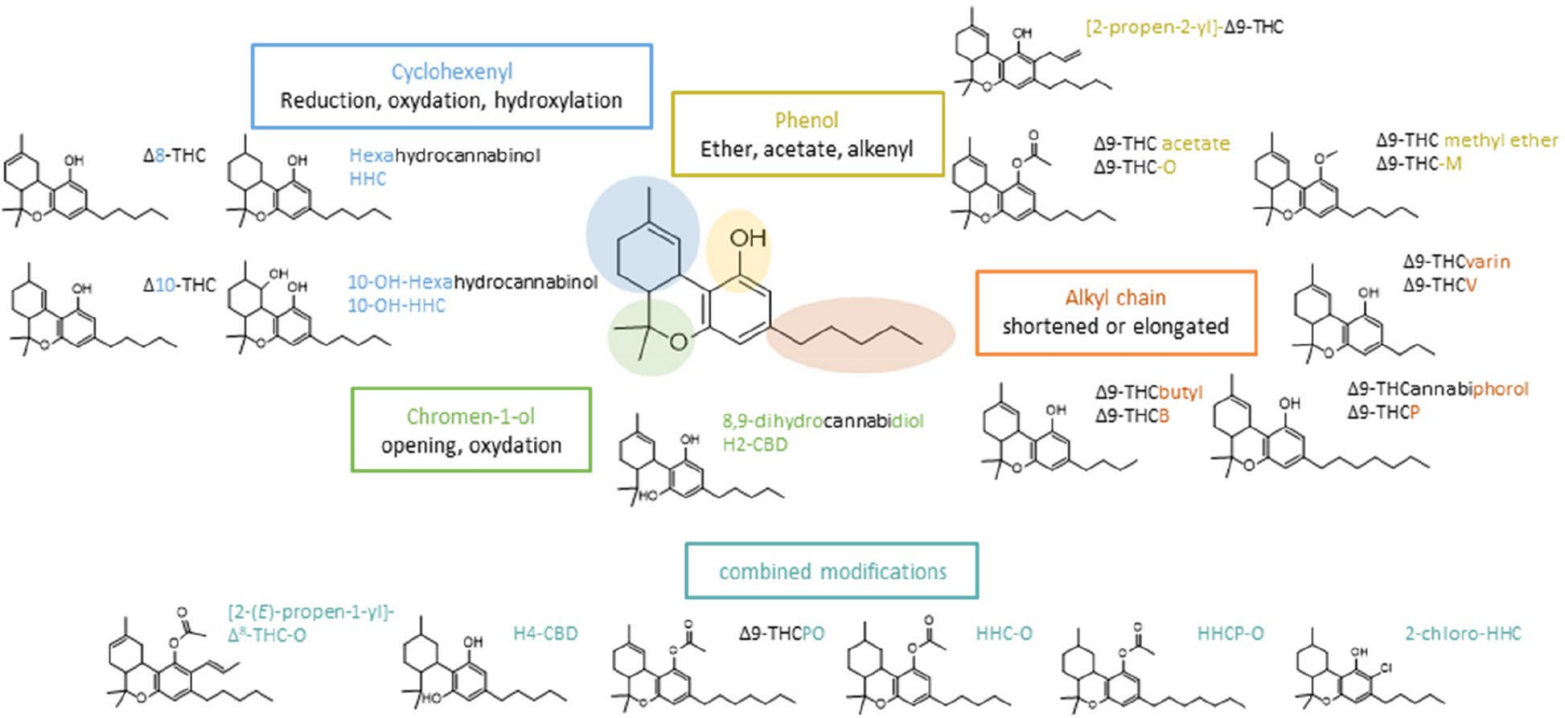
Semi-synthetic cannabinoids. Possible structural modifications compared to Δ^9^-THC on cyclohexyl, phenol, chromen-1-ol and/or alkyl chain functions leading to semi-synthetic cannabinoid derivatives

Among SSC (Fig. 1), hexahydrocannabinol (HHC), the hydrogenated analog of Δ^9^-THC, presents as two epimers (6a*R*,9*R*,10a*R*)-HHC (9*R*-HHC) and (6a*R*,9*S*,10a*R*)-HHC (9*S*-HHC); 9*R*-HHC being considered the most toxic one (Russo et al. 2023; Casati et al. 2024; Jørgensen et al. 2024; Greene et al. 2025; Šíchová et al. 2025). Although discovered in 1940, HHC appeared on the North-American drug market in 2021, was identified as a drug of abuse in May 2022 and was classified by the European Union Drugs Agency as an NPS in March 2023. Since 2021, HHC has been detected in various products, in particular in e-liquids and other vaping products (Casati et al. 2024; Jørgensen et al. 2024; Tanaka and Kikura-Hanajiri 2024; Ujváry 2024). Most of the literature on HHC focuses on its detection in biological samples from individuals suspected of drug-impaired driving (Bottinelli et al. 2024; Kronstrand et al. 2024; Höfert et al. 2024; Pitterl et al. 2024) or from study volunteers (Kobidze et al. 2024; Schirmer et al. 2023; Trana et al. 2021).

In the present study, we report a documented case of severe intoxication involving HHC in a male patient who developed severe rhabdomyolysis and an AKI, requiring a 15-day hospitalization. Although rare, severe intoxications involving seizure and hospitalization have been reported with other SSC (Iketani et al. 2024; Thomsen et al. 2025). Thanks to 17 blood samples collected over the entire hospitalization, the intoxication was investigated through an untargeted metabolomic approach combined to molecular networking. It enabled the identification of HHC metabolites, *i.e.*xenometabolome, confirming a mono-intoxication attributable to this SSC. In addition, characterization of the endometabolome made it possible to identify disturbed metabolic pathways by correlating endometabolites with HHC concentration and biochemical parameters of rhabdomyolysis followed by AKI.

## Case description

A 35-year-old male (160 cm, 80 kg; BMI, 31.3 kg/m^2^) with a history of congenital bilateral retinoblastoma was admitted to our intensive care unit (ICU) in a comatose state. In 2019, he presented a single epileptic seizure. He reports chronic tobacco use (40 pack-years) since adolescence, occasional CBD use and currently daily vaping of HHC, bought on the Internet. In the previous days, he experienced headaches, dizziness and impaired attention. At 10:00 on the day of admission (day 0, D0), he went outside to smoke. At 10:45 (considered as the time of exposure), he was found by his wife, alerted by an unusual dull noise, lying on the ground, unresponsive to verbal stimuli. She reported an initial presentation with generalized muscular rigidity, which progressed to tonic–clonic seizure involving all four limbs, further accompanied by urine incontinence and profuse hypersalivation. When the prehospital medical team arrived at home at 11:30, the patient was comatose (Glasgow Coma Scale score of 3) with stertorous breathing, lying on the floor in a postictal state. He promptly received clonazepam and oxygen and was transported to the hospital at 12:45.

**Fig. 2:**
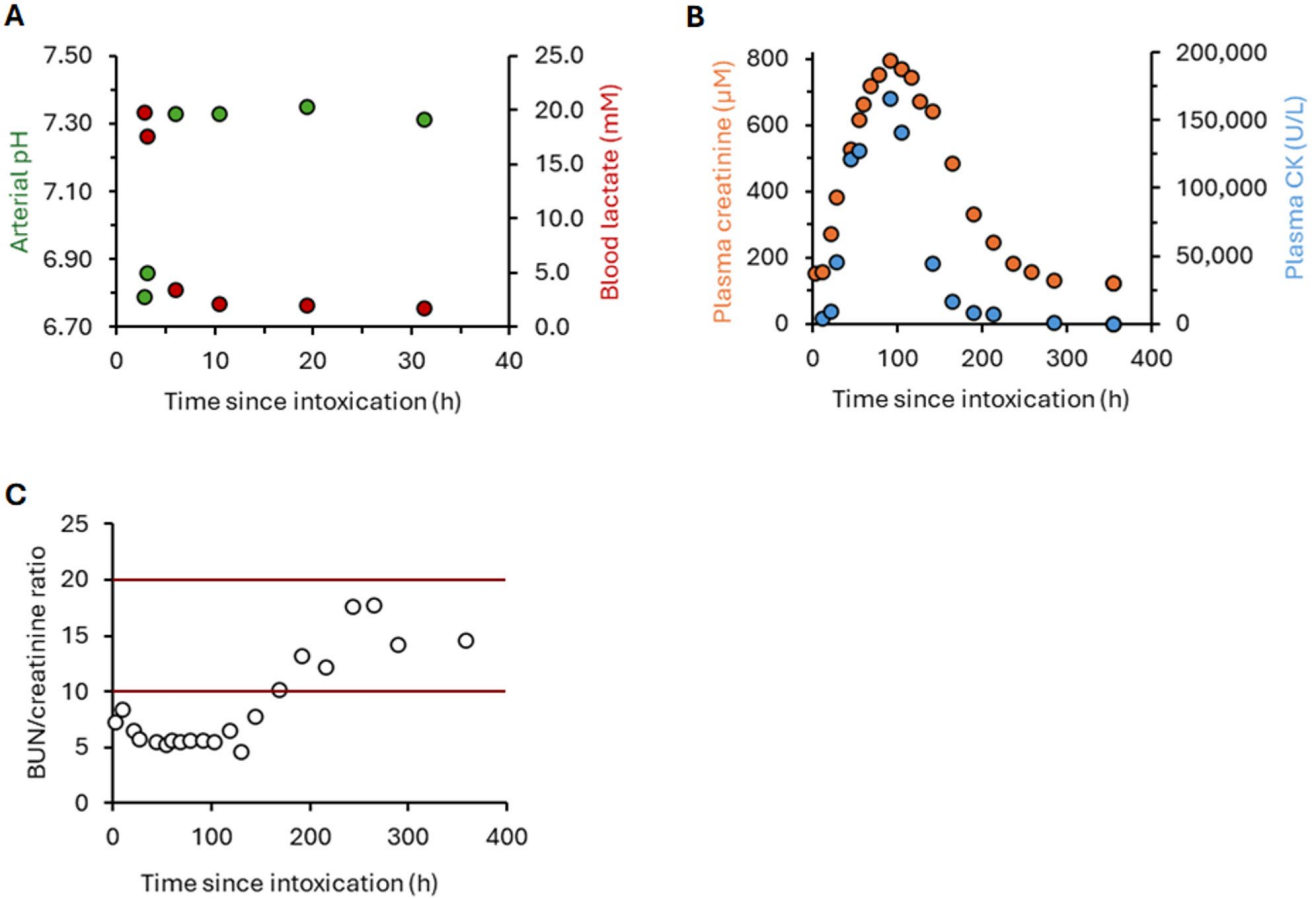
Time-course of the biochemical parameters. The patient was admitted to the ICU with a transient severe lactic acidosis (A), followed by rhabdomyolysis-induced acute kidney injury, as evidenced by the increase in plasma creatinine and creatine kinase (CK) (B) associated with the decrease in blood urea nitrogen (BUN)/plasma creatinine ratio (C). All metabolic and kidney dysfunctions observed in this poisoned patient progressively recovered with supportive care.

On admission, CT-scan showed a minor right frontal subarachnoid hemorrhage without ventricular involvement and a T12 vertebral fracture, both attributed to the post-epileptic fall. Electrocardiogram showed sinus tachycardia (150 bpm) without additional abnormalities. Transient lactic acidosis (arterial pH, 6.80; blood lactate, 19 mmol/L) was assessed, which rapidly reversed (pH, 7.33; blood lactate, 3 mmol/L) as soon as 17:00 (Fig. 2.A). Levetiracetam was infused to prevent seizure recurrence, along with fluid and acyclovir to treat a potential herpesvirus simplex meningoencephalitis, as his body temperature was 38.2 °C and his cerebrospinal fluid hemorrhagic (due to subdural hematoma), difficult to interpret. Further chemistry tests showed rhabdomyolysis (creatine phosphokinase, 4,200 IU/L on D0, peaking at 166,000 U/L on D4; normal range, 55–170 and serum myoglobin, > 10,000 µg/L; reference range, 25–72) and subsequent AKI (oliguria with serum creatinine peaking at 796 µmol/L on D4; normal range, 64–115) (Fig. 2.B). Serum aspartate (AST) and alanine aminotransferases (ALT) peaked at eight- and 40-fold the upper limit on D4, respectively.

**Fig. 3:**
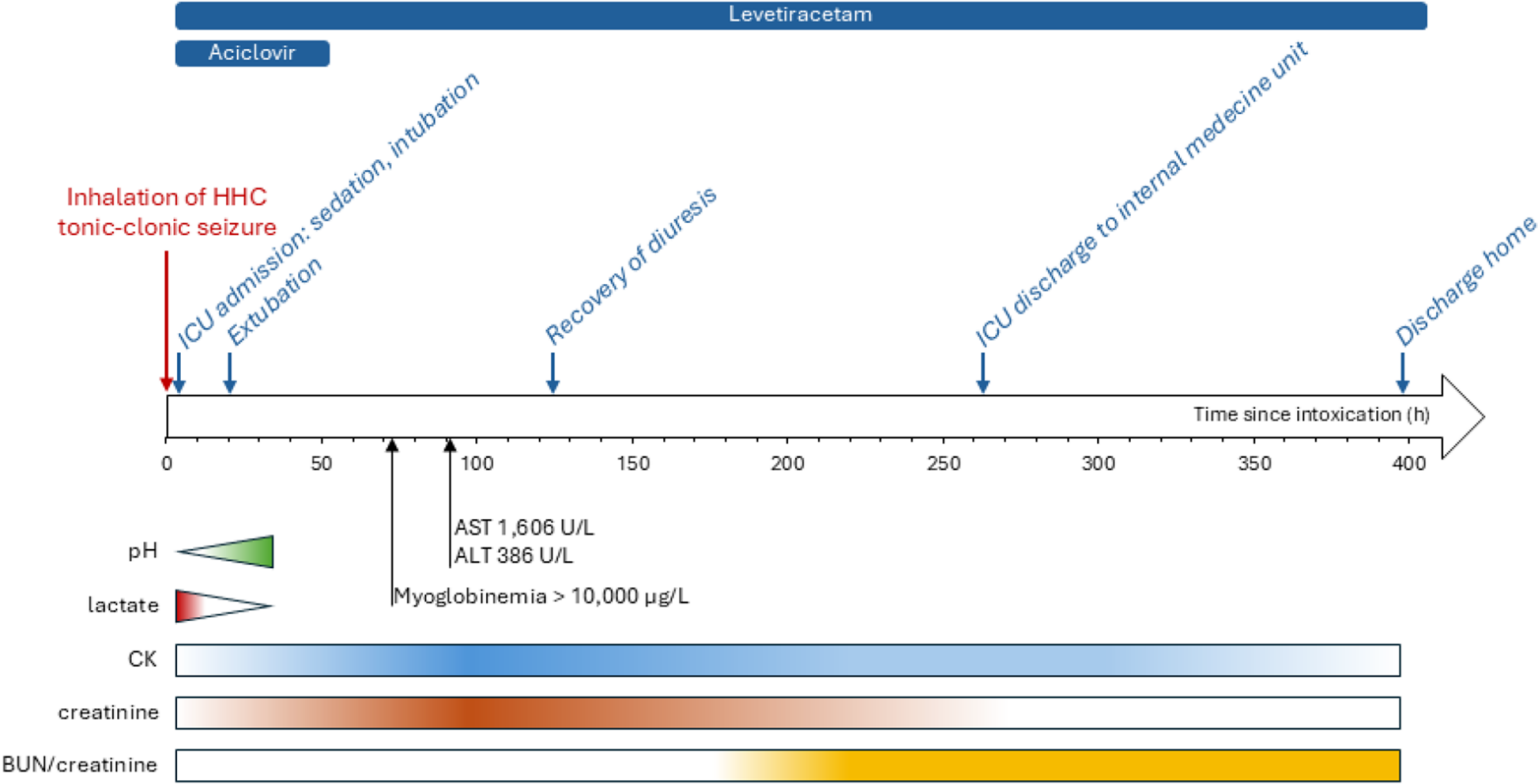
Evolution of the patient's clinical and biological parameters during hospitalization. The patient initially presented a lactic acidosis, followed by a rhabdomyolysis-induced acute kidney injury peaking at D4. Supportive management led to the progressive recovery of renal function. ALT, alanine aminotransferase; AST, aspartate aminotransferase; BUN, blood urea nitrogen; CK, creatine kinase

Rapidly, the patient improved. He was extubated on D1, and acyclovir was discontinued following negative microbiological results and the absence of clinical signs of infection. His diuresis and renal function gradually recovered without dialysis. His blood urea nitrogen(BUN)/serum creatinine ratio decreased before normalizing one week later (Fig. 2.C). Biological and medical evolution of the patient during his hospitalization are described in Fig. 3. He was discharged from the ICU and transferred to the medical ward on D11.

Toxicological screening performed on admission showed the presence in plasma of cotinine, in addition to levetiracetam, rocuronium and etomidate administered as treatments. Urine immunoassay drug testing was positive for 11-nor-9-carboxy-Δ^9^-tetrahydrocannabinol (THC-COOH) (Siemens Syva® Emit® II Plus Cannabinoid), a metabolite typically detected after Δ^9^-THC use. Seventeen plasma samples together with three urine samples were collected during his stay for additional measurements. This laboratory study, was conducted in accordance with the Helsinki Declaration as part of an ongoing clinical investigation on the pharmacokinetic-pharmacodynamic relationships in psychotropic drug poisonings, approved by the ethics committee of the French Society of Intensive Care (protocol number, FICS20020231). An additional evaluation was performed on six vaping products, reportedly used by the patient and provided by his wife.

## Materials and methods

### Chemicals and reagents

9*R* and 9*S*-HHC, 11-nor-9(*R*)-carboxy-HHC (9*R*-HHC-COOH), Δ^9^-THC, 11-hydroxy-Δ^9^-THC (THC-OH), THC-COOH, THC-COOH-glucuronide, cannabidiol (CBD), 7-hydroxycannabidiol (CBD-OH), 7-carboxy-cannabidiol (CBD-COOH), cannabinol (CBN), Δ^9^-THC-D3, THC-OH-D3, THC-COOH-D3, CBD-D3, CBD-OH D3, CBD-COOH-D3 and methadone-D3 were purchased from LoGiCal® (LGC GmBH, Molsheim, France). *Tert*-butyl-methyl-ether (MTBE) and dichloromethane were supplied by Sigma-Aldrich (Saint-Quentin Fallavier, France). High performance liquid chromatography—mass spectrometry—grade methanol (MeOH), acetonitrile (ACN), isopropanol (IPA), water and formic acid (FA) were purchased from Fisher Scientific (Courtaboeuf, France). Blank human plasma was purchased from the French Blood Establishment (EFS, Paris, France). Blank urine was obtained from a drug-free volunteer laboratory employee and tested before use.

### E-liquids and vape products analysis

#### Sample preparation

Vaping product tanks, mostly empty, were rinsed with MeOH. All samples were then extracted with 2.0 mL of MTBE after addition of 200 µL of phosphate buffer (100 mM, pH 2). Following a centrifugation step, supernatant was evaporated under a gentle steam of nitrogen and dry extracts were dissolved in 100 µL of ACN. A volume of 1 µL was injected into the GC–MS analytical system.

#### Instrument conditions

The GC–MS system was based on a Trace 1310® hyphenated to an electron impact—ISQ LT® (Thermo Fisher Scientific). All technical details are provided in Additional File 1.

#### Data processing

System control, data acquisition and processing were carried out using Xcalibur software (Thermo Fisher Scientific). Compound identification was performed by comparing the mass spectra to the following international libraries: NIST, Cayman, SWGDrug 3.14.

### Plasma and urine samples preparation

All biological samples were kept at −20 °C and analyzed in a single batch at the end of hospitalization. One urine sample was extracted twice, with and without albalonase deglucuroconjugation (*v/v*, 30 min at 60 °C). Enzymatic deconjugation was deliberately not applied to the remaining samples for two reasons: first, to allow the independent evaluation of phase I and phase II metabolite kinetics; and second, because enzymatic deconjugation of cannabinoid metabolites is known to be incomplete and often requires an additional alkaline hydrolysis step. The consequences of such chemical treatment on the metabolites of interest are unknown in our case, and we therefore chose not to perform it. Briefly, 100 µL of biological matrix/QC/calibrant was aliquoted in Eppendorf, 150 µL of phosphate buffer (100 mM, pH 2), 350 µL of ACN containing IS and 350 µL of IPA were added. Samples were vortexed for 1 min, kept at −20 °C during 15 min for protein precipitation and centrifuged at 18,600 g for 10 min. Supernatant was transferred in a 5 mL glass tube and evaporated under a gentle flux of nitrogen. Dried extracts were reconstituted in 100 µL of MeOH/water (50/50, *v/v*), vortexed for 1 min, centrifuged at 2,500 g for 5 min and transferred in an insert vial. Prepared samples were then injected three times in the UHPLC-ESI^±^-HRMS system according to the conditions mentioned for quantitative targeted analysis and xenometabolomic and endometabolomic investigations. Prior to the injection, QC for metabolomic analysis was prepared by an equivolumetric mixture of each patient plasma extract. This QC was then injected periodically throughout the two metabolomic analysis.

### Hair sample preparation

Briefly, a brown hair sample (5 cm) cut from the posterior vertex region during his ICU stay was washed with dichloromethane before separation in two proximal and distal segments of 2.5 cm. Once microsegmented, milled and weighted, 1 mL of MeOH containing THC-D3 at 5.0 ng/mL were added. Samples were agitated 1 h, centrifuged 10 min at 5,000 rpm and supernatant was evaporated to dryness at 40 °C under a gentle flux of nitrogen. One hundred microliter of MeOH/water (50/50, *v/v*) were added to the dried extract, vortexed and 5 µL were injected into the UHPLC-ESI^+^-HRMS using parameters mentioned below for a quantitative targeted analysis. Method was validated according the ICH guidelines, and calibration was performed from 5 pg/mg (Limit of quantification LOQ) to 1,000 pg/mg of hair, with spiked hair previously tested for cannabinoids.

### Quantitative targeted analysis

Quantitative plasma targeted analysis was performed using a method validated according to the ICH guidelines, including accuracy and precision within 15% of the targeted value.

#### Calibration solutions and quality control preparation

Two stock solutions of cannabinoids, one for calibration and one for quality controls, containing 9*R*-HHC, 9*S*-HHC, 9*R*-HHC-COOH, Δ^9^-THC, THC-OH, THC-COOH, THC-COOH-glucuronide, CBD, CBD-OH, CBD-COOH and CBN were prepared at 10.0 µg/mL in MeOH and stored at −20 °C. Deuterated IS stock solution containing THC-D3, THC-OH-D3, THC-COOH-D3, CBD-D3, CBD-OH-D3, CBD-COOH-D3, methadone-D3 at 10.0 ng/mL was prepared in ACN and stored at −20 °C. Calibration solutions were prepared extemporaneously by diluting the stock solution in blank plasma, to obtain eight cannabinoid concentration solutions: 0, 0.5, 1.0, 2.0, 5.0, 10.0, 50.0, 100.0 and 200.0 ng/mL. Quality control samples (QC) were prepared in parallel of the calibration levels using the second stock solution of cannabinoids and further diluted in blank plasma to obtain three concentrations: 1.5 (QC1), 15.0 (QC2) and 150.0 (QC3) ng/mL.

#### Instrument conditions

Five microliters of prepared samples were injected into the UHPLC-ESI^+^-HRMS system, constituted of a Vanquish® UHPLC hyphenated to a QExactive Focus® mass spectrometer from Thermo Fisher Scientific (Les Ulis, France). Column, gradient, and source parameters are provided in Additional File 1 and were specifically optimized for phytocannabinoid analysis, allowing separation of the 9*R* and 9*S* diastereoisomers of HHC (Additional File 2).

#### Data processing

System control, data acquisition and processing were carried out with TraceFinder Forensic 4.1® software (Thermo Fisher Scientific).

### Metabolomic analysis

#### Instrument conditions

For the xenometabolomic analysis, samples and QC were injected in the UHPLC-ESI^+^-HRMS as for the quantitative targeted analysis, *i.e. *identical chromatography column and gradient, but with different source and scan parameters (see Additional File 1). For the endometabolomic analysis, 10 µL of extracted samples or QC were injected on the same UHPLC-ESI^±^-HRMS system previously mentioned, but with a different method provided in Additional File 1.

#### Data processing

Acquisition of data was carried out with TraceFinder Forensic 4.1® software. Raw data files were then processed according to an in-house workflow, previously described (Magny et al. 2024). Briefly, after conversion into open-source mzXML files, data were processed using MZmine 3.9.0. Critical parameters were the same as mentioned in the publication source, except for the following ones: mass detection noise level was set at 1E4 for MS spectra and 1E3 for MS/MS spectra, minimum search range RT/mobility was 0.05 and peak duration range 0.2 for local minimum feature resolver, and 0.1 min for join aligner. Extracted ion chromatograms were built using the ADAP algorithms. The ADAP wavelets chromatogram deconvolution algorithm was then applied before de-isotope grouping, peak alignment and gap filing processing. Annotation of metabolites was obtained using the GNPS database library, an in-house database, MS and MS/MS spectra analysis, supported by SIRIUS 5.4 for molecular formula determination and metabolites annotation. Molecular networking modeling was carried out with MetGem software, with parameters as previously published (Magny et al. 2023).

The semi-quantification was based on the use of IS during sample preparation: intensities of each metabolite were normalized individually to the THC-COOH-D3 intensity for HHC metabolites analysis. For endogenous metabolome, normalization was performed with methadone-D3 and CBD-COOH-D3 in positive and negative ion mode respectively. Then, normalized ratios were related to the ratio determined in the admission sample for evaluation of the time concentration evolution. Following the normalization step, only the annotated metabolites with a CV% in the QC samples below 30% were retained for the rest of the endometabolome analysis, reducing the dataset from an initial 2,995 variables to a total of 1,341 variables. The annotation step was performed by querying in-house and GNPS database libraries, followed by manual verification prior to validation. Duplicate features were removed, resulting in a total of 145 identified endogenous metabolites.

### Statistical analysis

Figures and statistics were performed using PRISM 10.2 software® (GraphPad Software, La Jolla, USA). We employed the Spearman rank correlation test. Given the large number of comparisons performed, we applied the Benjamini–Hochberg false discovery rate (FDR) correction to control for type-I error, while maintaining statistical power. Significance is notified as * *p* < 0.05, ** *p* < 0.01, *** *p* < 0.001, **** *p* < 0.0001.

## Results

### Vaping products

Two vape products contained CBD, CBN, cannabielsoin and cannabicitran. Three other vape products contained only HHC with no other cannabinoid. The last vaping product contained CBD, HHC and trace amounts of HHC-acetate. As e-cigarette tanks were mostly empty, the remaining liquid was extremely difficult to sample due to the fluid viscosity. HHC concentration in the MeOH that was used to rinse tanks was estimated at 100 µg/mL in two of the vaping products. Considering that the vaping liquid probably represented ≈ 10% of the volume sampled after the rinse with MeOH, results are in accordance with the packaging of the vape products that claims to contain 85% of HHC.

### Quantitative targeted analysis

**Fig. 4:**
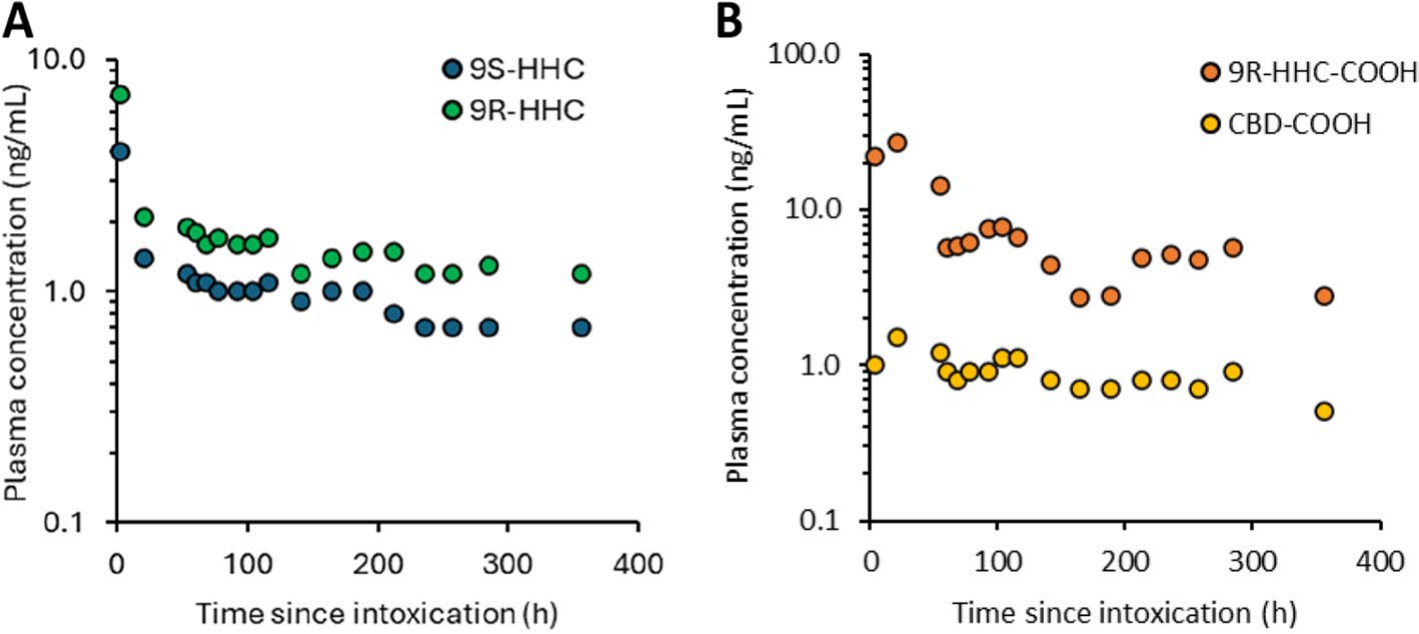
Plasma cannabinoid concentrations. Native HHC (9*R* and 9*S* epimers) concentration, monitored during the 15 days of hospitalization, decreased rapidly to reach a plateau value (**A**). Carboxylic metabolites of HHC and CBD peaked at 24 h and remained detectable on D15 (**B**)

Cannabinoids were quantified in 17 plasma samples obtained over the 15 days of hospitalization. Δ^9^-THC and its metabolites, CBD, CBD-OH and CBN were undetectable (limit of detection LOD, 0.2 ng/mL). 9*R* and 9*S*-HHC concentrations rapidly decreased during the first hours post-exposure, from 7.1 to 1.2 and from 4.0 to 0.7 ng/mL and remained detectable in the plasma after 15 days (Fig. 4A). 9*R*-HHC-COOH and CBD-COOH were quantified in the admission plasma sample (H3) at 22.1 and 1.0 ng/mL, respectively. They increased during the first 24 h before decreasing but were still detectable after 15 days (Fig. 4B). On ICU admission, urine 9*R*-HHC-COOH concentrations were 8 and 217 ng/mL before and after deglucuronidation, respectively. Absolute quantification of the other HHC metabolites was not performed since commercial standards were not available at the time of analysis. Hair sample analysis indicated the presence of HHC as 9*R* and 9*S* epimers at < LOQ and 7.0 pg/mg in the proximal segment, and at 6.4 and 18.9 pg/mg in the distal segment respectively.

### Metabolomic analysis

#### Identification of HHC metabolites

**Fig. 5:**
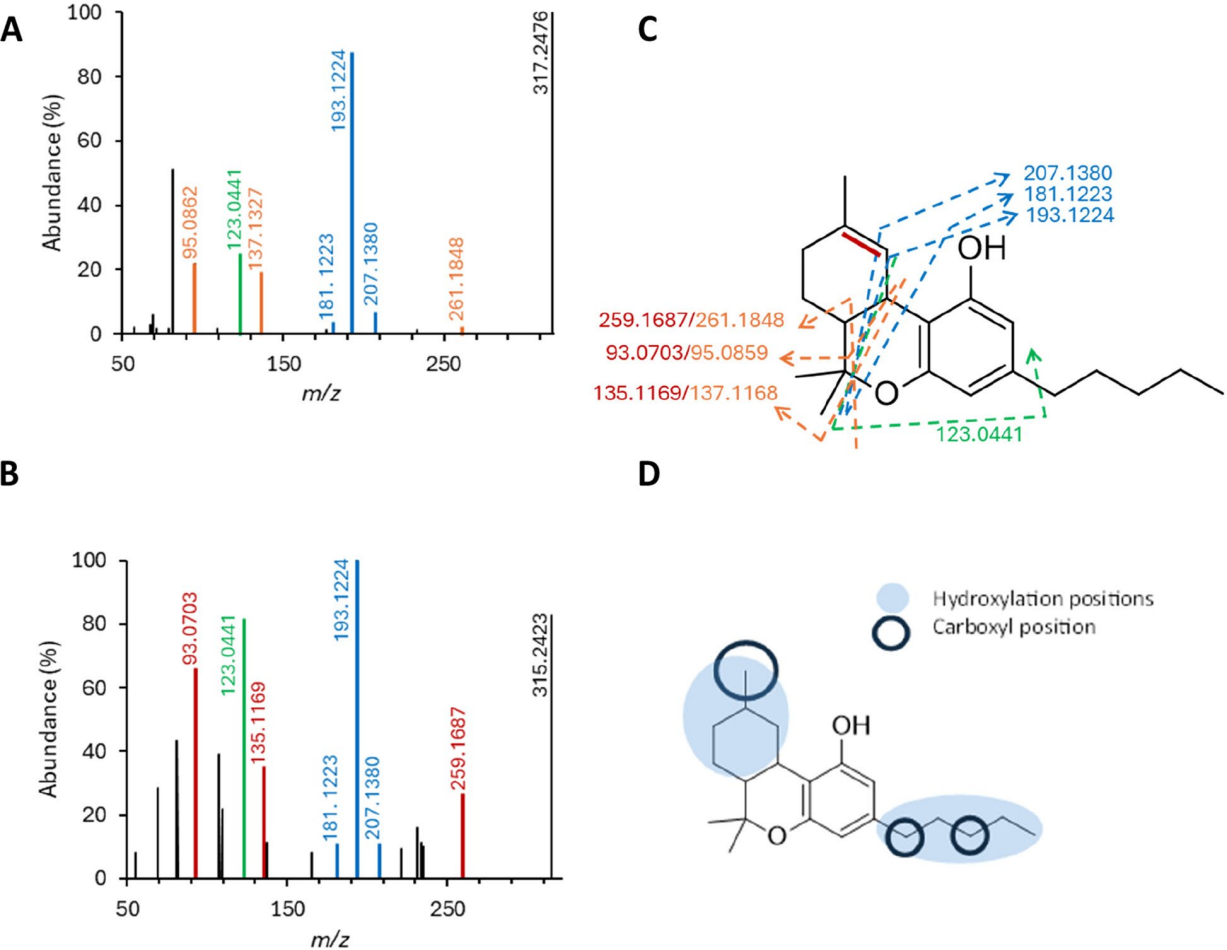
MS/MS spectra of 9*R*-HHC and Δ^9^-THC and proposed fragmentation pattern. ESI^+^ MS/MS spectra of 9*R*-HHC (**A**) and Δ^9^-THC (**B**) display common and specific product ions, enabling to propose a fragmentation pattern (**C**): 2-methylresorcinol core shared by all phytocannabinoids (), combined with pentyl chain () and specific fragments of 9*R*-HHC () and of Δ^9^-THC (). Phase I HHC metabolite structures were determined based on the same fragmentation pattern (**D**)

**Fig. 6:**
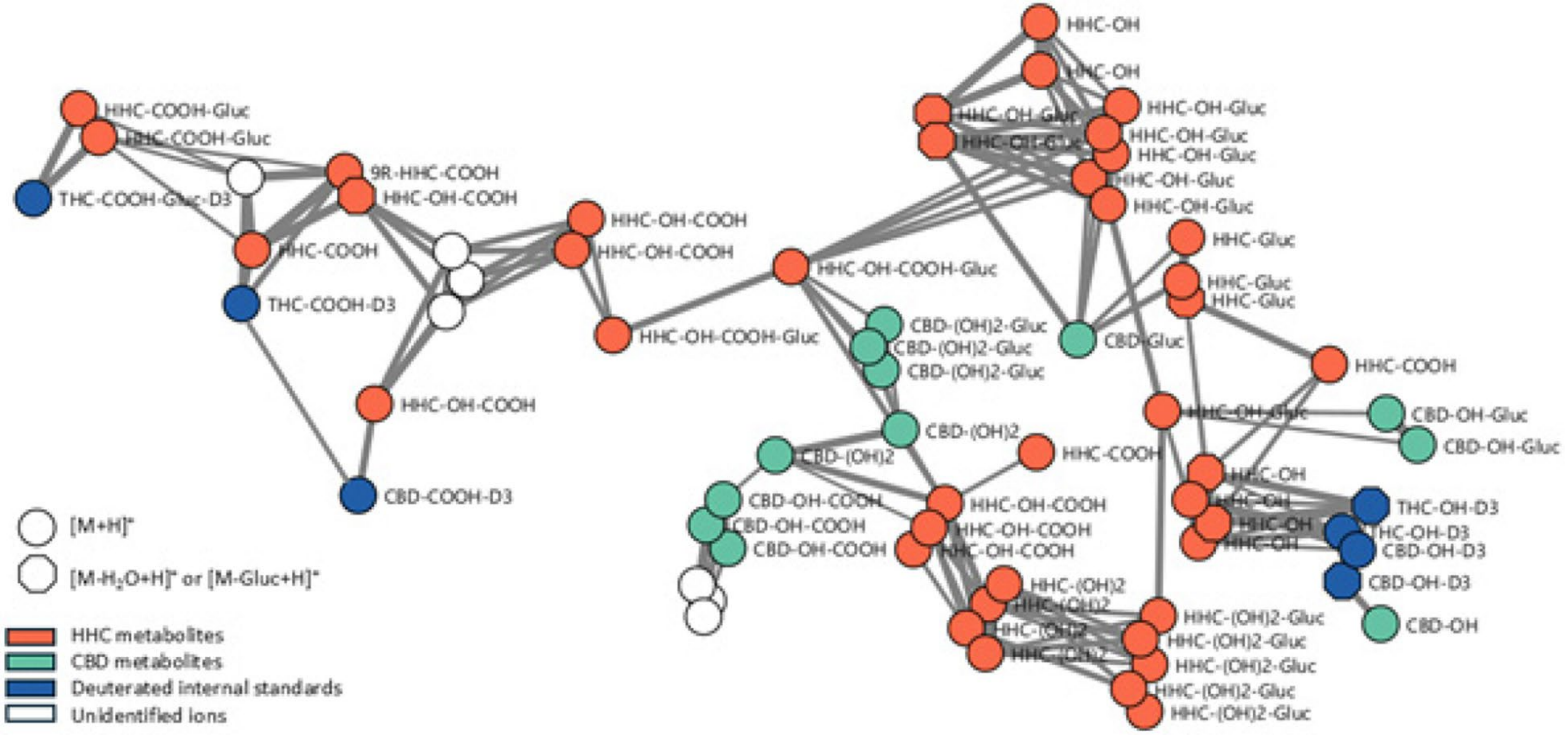
Molecular network of cannabinoids based on MS/MS data acquisition. A total of 35 HHC metabolites () and 12 CBD metabolites () were identified in the patient’s biological samples. The network was anchored by means of deuterated commercial standards of cannabinoids ()

Data processing of the biological extracts analyzed using untargeted UHPLC-ESI^+^-HRMS along with molecular network implementation led to the annotation of 35 HHC metabolites, including 17 glucuroconjugated ones (Additional File 3). Comparison of MS/MS spectrum of HHC and Δ^9^-THC allowed proposing a detailed fragmentation pattern (Fig. 5) and elucidating HHC metabolites structures obtained from the generated molecular network (Fig. 6).

Annotation of glucuroconjugated metabolites was based on the detection of *i*) the neutral loss of the glucuronide radical, *i.e.* a mass shift of 176.0309, *ii*) the product ion corresponding to the aglycone, *i.e.* the phase I metabolite and *iii*) specific product ions of the 2-methylresorcinol, methylcyclohexyl and pentyl parts. Of note, two nodes corresponding to HHC-glucuroconjugates were identified in the molecular network. They displayed the same MS/MS spectra but 9*S*-HHC and 9*R*-HHC were chromatographically identified using the developed conditions. Thus, these glucuronide metabolites likely corresponded to the epimers of HHC. Four HHC-OH, four HHC-(OH)_2_, six HHC-OH-COOH and four HHC-COOH were identified with [M + H]^+^ parent ion and [M + H-nH_2_O]^+^ fragment ion(s) (n = 1 and/or 2).

**Fig. 7:**
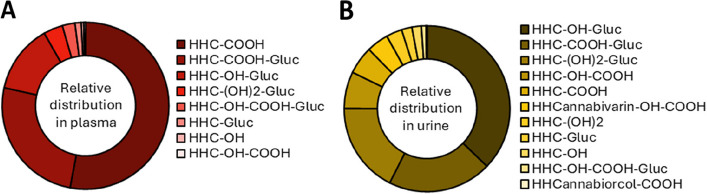
Relative distribution of HHC metabolites in plasma and urine. Isomeric metabolites were grouped together, and their relative distributions based on signal intensity in ESI.^+^ are shown for the admission plasma (**A**) and urine (**B**) samples collected at H3

Regarding the hydroxylated metabolites, MS/MS spectra were useful to probe the position where the reaction had occurred. Product ions at *m/z* 193.1224, 181.1223 and 207.1380, detected in both HHC and Δ^9^-THC MS/MS spectra, were indicative of an unmodified lateral pentyl chain. Consequently, the presence of these product ions in the MS/MS spectrum suggested that the hydroxyl and carboxyl groups were most likely located on the methylcyclohexyl ring—specifically at positions C8/C9/C10 (both epimers) or C11. Furthermore, the product ions at *m/z* 191.1063, 179.1060 and 205.0859 suggested the presence of an unsaturation on the lateral pentyl chain, likely resulting from dehydration, and were therefore indicative of hydroxylation on this side-chain (Additional File 3). As the 35 identified HHC metabolites shared relatively similar structures, it was reasonable to assume that they exhibited comparable ionization efficiencies in ESI^+^ mode. Accordingly, for each sample collected during hospitalization, we compared their signal intensities to determine which metabolites were predominantly present in urine and plasma on admission (Fig. 7).

**Fig. 8:**
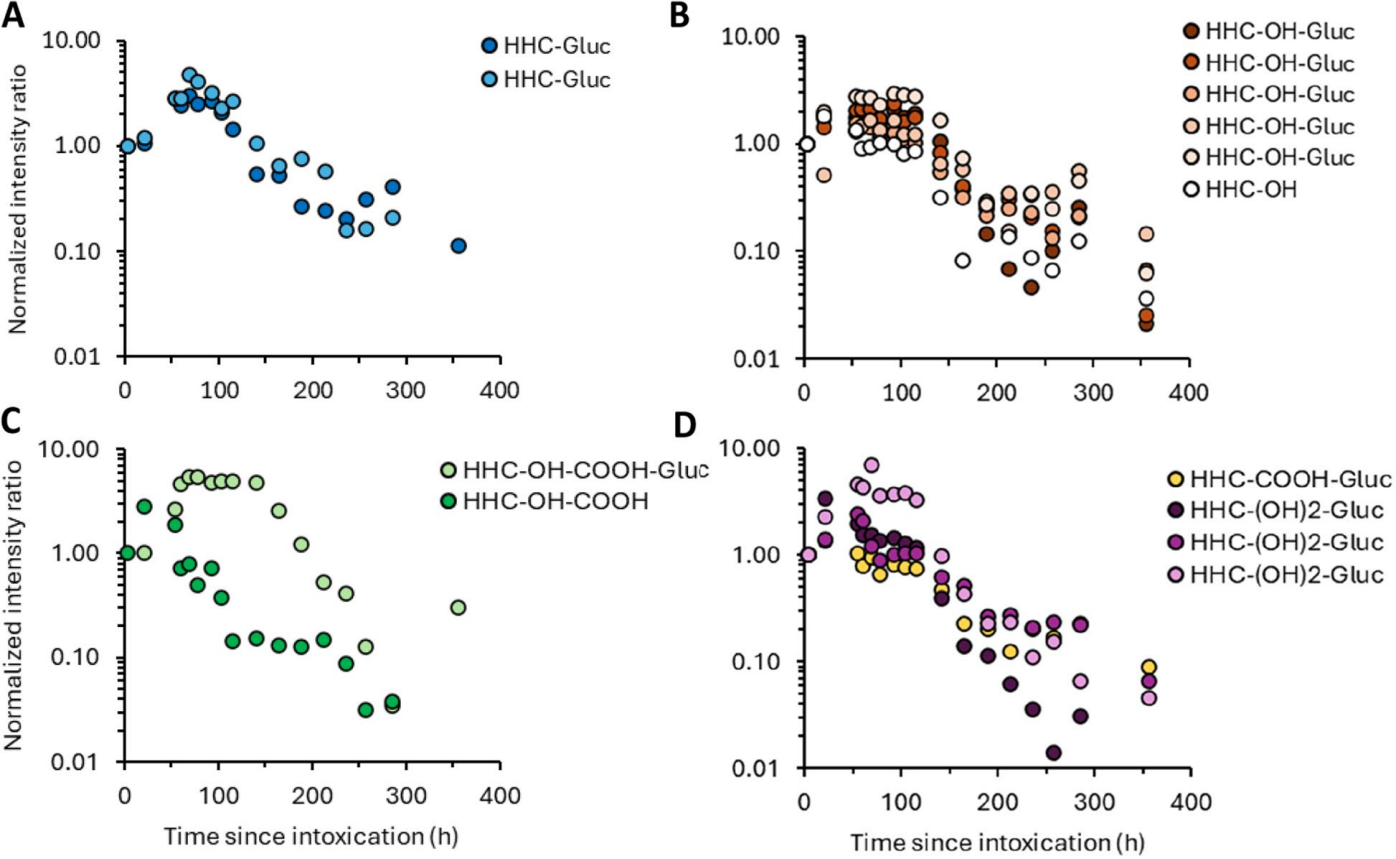
Plasma kinetics of HHC metabolites, based on semi-quantification. Time course of HHC-glucuronides (**A**), HHC-OH and glucuronides (**B**), HHC-OH-COOH and glucuronide (**C**), HHC-(OH)2-glucuronides and HHC-COOH-glucuronide (**D**). Metabolite level is displayed as normalized intensity ratio to the first plasma sample

Although HHC-OH-glucuronides were the most abundant metabolites in urine, HHC-COOH and its glucuronide represented more than 75% of the metabolites in plasma. The kinetics of each identified metabolite were assessed in plasma over the l5-day hospitalization period (Fig. 8). While glucuronide metabolites peaked approximately 100 h after exposure, phase I metabolites peaked within the first 24 h. Twelve metabolites were detectable in plasma when the patient was discharged from the hospital. Cerebrospinal fluid, although hemorrhagic, presented a concentration of 9*R*-HHC-COOH at 9 ng/mL, while other cannabinoids were undetectable.

#### Endometabolome analysis

Based on an untargeted analysis and the identification of 145 molecules including amino acids, organic acids, vitamins and lipids, three correlation matrices were built in relation to plasma 9*R*-HHC, serum creatinine and serum CK concentration. However, due to the fewer CK compared to creatinine values (10 *vs*. 17), some metabolites exceeded the threshold of statistical significance. All matrix correlations are presented in Additional File 4.

Among the identified metabolites, 20 significantly correlated with 9*R*-HHC concentrations, including phenylalanine (r = 0.83, *p* = 0.0017), phenylpyruvic acid (r = 0.83, *p* = 0.0019), kynurenic acid (r = 0.73, *p* = 0.0147), citrate (r = 0.72, *p* = 0.0148) and hippurate (r = 0.64, *p* = 0.0498).

The patient presented rhabdomyolysis along with AKI, with serum CK and creatinine peaking at D4. CK and creatinine were strongly correlated (r = 0.98, *p* = 0.0003). Several metabolites were also significantly correlated with creatinine including valine (r = −0.71, *p* = 0.0094), tryptophane (r = −0.71, *p* = 0.0087), taurine (r = 0.75, *p* = 0.0050), kynurenic acid (r = 0.78, *p* = 0.0026), guanine (r = 0.80, *p* = 0.0017) and dimethylguanosine (r = 0.86, *p* = 0.0003). So did six diacids including azelaic acid (r = 0.81, *p* = 0.0011), 3-methyladipic acid (r = 0.84, *p* = 0.0005), suberic acid (r = 0.77, *p* = 0.0030), sebacic acid (r = 0.84, *p* = 0.0005), tetradecanedioic acid (r = 0.82, *p* = 0.0008) and dodecanedioic acid (r = 0.66, *p* = 0.0167). Several lipids were also correlated with creatinine including carnitine (r = −0.90, p < 0.0001) and several medium- and long-chain acylcarnitines including lauroylcarnitine (r = 0.61, *p* = 0.0366), tetradecanoylcarnitine (r = 0.59, *p* = 0.0420) and O-2-tetradecanoylcarnitine (r = 0.60, *p* = 0.0371). Finally, vitamin B1 metabolism appeared to be related to the rhabdomyolysis/AKI processes, since thiamine (r = 0.94, p < 0.0001), 5-(2-Hydroxyethyl)−4-methylthiazole (r = 0.95, p < 0.0001), 4-pyridoxate (r = 0.91, p < 0.0001) and pyridoxine (r = 0.84, *p* = 0.0005) were significantly correlated with plasma creatinine and plasma CK concentrations.

## Discussion

Although SSCs have been present on the market for years, their increasing prevalence in cannabis-derived products raises major public health concerns due to their known harmful effects (Casati et al. 2024; Jørgensen et al. 2024; Tanaka and Kikura-Hanajiri 2024; Ujváry 2024). Besides neurological, cardiovascular and gastrointestinal complications, SC exposure can result in rhabdomyolysis and, regardless of the underlying mechanism, in AKI (Bosch et al. 2009). In the US, rhabdomyolysis is most frequently associated with amphetamines, while in Europe, it is more commonly linked to SC (Amanollahi et al. 2023). To our knowledge, no such cases have yet been reported for SSCs, although severe intoxication involving seizure have been described recently (Iketani et al. 2024; Thomsen et al. 2025). Among SSCs, HHC used to be widely and legally distributed, but became more recently prohibited in most countries in response to growing safety concerns. In the present study, we report a case of severe HHC poisoning resulting in seizure, massive rhabdomyolysis and AKI. A set of biological samples analyzed using UHPLC-ESI^±^-HRMS in a non-targeted metabolomic approach combined with molecular networking, enabled to obtain insights into HHC kinetics and biochemical pathways disrupted by HHC exposure.

### Identification of the toxicant

Three sources of information contributed to the toxicant identification. The first source was the history reported by the patient's wife and later confirmed by the patient, *i.e.,* the use of products only containing cannabis or SSCs. The second source was the analysis of the 1–2 mL of vaping products he had consumed, as provided on-site by his wife. Two vape products were labelled as containing 85% CBD and three others as containing HHC (85% for one). The vape products labeled CBD were found to contain a large content of CBD and several natural phytocannabinoids including cannabinol, cannabielsoin and cannabicitran suggesting that these products were manufactured from plant extracts. The vape products labeled HHC exclusively contained HHC, in equal proportions of its two epimers, 9*R* and 9*S*, indicating a synthetic origin. Given the absence of other psychoactive substances, the poisoning could be attributed to one or both cannabinoids identified, namely CBD and HHC. The third identification source was the biological sample analysis. An immunoassay for urinary cannabinoids on admission yielded a positive result, and toxicological screening ruled out the presence of any other psychoactive substances. An additional analysis selectively focused on cannabinoids was in line with the information provided by the patient’s wife and the results of the analysis of the vaping e-liquids, showing that neither Δ^9^-THC nor any of its metabolites were detected in the biological samples. The positive result of the urinary cannabinoid immunoassay is not inconsistent with these findings, as it simply reflects the assay’s lack of specificity. Regarding CBD, UHPLC-ESI^+^-HRMS analysis identified several CBD metabolites in the urine, as well as its terminal metabolite, CBD-COOH, in the plasma. The presence of the latter in trace amounts, along with the absence of detectable CBD, confirms prior CBD use but suggests that it occurred well before the epileptic event, thereby supporting the exclusion of this cannabinoid as a contributing factor. Interestingly, a relatively high plasma HHC concentration (11 ng/mL) was assessed on admission.

Accordingly, we implemented an analytical strategy aimed at achieving the most accurate and exhaustive annotation of phase I and phase II HHC metabolites, expected to encompass a wide range of chemical structures, thus representing a remarkable challenge from an analytical toxicology perspective (D’Errico et al. 1936; Znaleziona et al. 2015). This non-targeted analytical strategy relied on an advanced system combining UHPLC-ESI^+^-HRMS, considered gold standard for its sensitivity and specificity (Magny et al. 2023, 2022). Analysis of chromatographic and low/high-energy mass spectrometric data combined with the use of HHC reference standards and available metabolites, allowed identifying 35 HHC metabolites based on molecular network construction. Fragmentation pattern elucidation was supported by previous studies on Δ^9^-THC and CBD (Linciano et al. 2020; Lelario et al. 2021). Among the identified metabolites were hydroxylated and carboxylated derivatives, as well as glucuronide conjugates, corresponding to phase I metabolism mediated by cytochrome P450 (CYP) 3A4, CYP2C9 and CYP2C19 isoforms, and phase II metabolism involving UDP-glucuronosyltransferases (Stout and Cimino 2014; Dinis-Oliveira 2016). This metabolic profile closely resembles those previously reported for Δ^9^-THC and CBD (Huestis 2005). Of note, the additional oxidative metabolites included dihydroxylated compounds and structures resulting from combined hydroxylation and decarboxylation, which have been described with Δ^9^-THC and recognized as markers of recent exposure (McBurney et al. 1986; Gasse et al. 2018). We also detected the presence of a metabolite recently reported by Pitterl et al. (Pitterl et al. 2024). Finally, molecular networking based on structural similarity of a large HHC metabolite number enabled to reconstruct a comprehensive picture of the metabolic fate of HHC in our patient. Of interest, most metabolites remained detectable in plasma up to fifteen days after exposure. This prolonged detectability supported a profile of regular—conceivably chronic—heavy HHC use, also evidenced by the detection of HHC in both proximal and distal segments of hair sample, indicative of a consumption for at least 3 months (Cuypers and Flanagan 2018). Such sustained and intense HHC use was likely associated with the severity of poisoning.

Based on the reference substances 9*R*-HHC, 9*S*-HHC and 9*R*-HHC-COOH, we quantified the two HHC epimers and key metabolite, and established their kinetic profiles over the entire hospitalization period. On admission, 3 h after seizing, plasma HHC concentration was measured at 11 ng/mL then decreased rapidly before stabilizing at 1–2 ng/mL over the subsequent 15 days. The shift in kinetic profiles between 9*R*-HHC and 9*S*-HHC, characterized by a more rapid decrease in 9*S*-HHC concentration, is consistent with previous findings (Trana et al. 2021). Detection of HHC in blood 15 days post-exposure highlights a kinetic profile similar to that of Δ^9^-THC, known to persist in chronic users for up to 30 days at low concentrations (Sempio et al. 2021; Bergamaschi et al. 2013). Similarly to Δ^9^-THC, prolonged HHC elimination, especially in chronic users like our overweight patient, is related to its high lipophilicity (log *P* of 7.72 *versus* 7.26 for Δ^9^-THC and HHC, respectively), which promotes their accumulation in adipose tissue and their gradual sustained release into the bloodstream in chronic users (Kreuz and Axelrod 1973; Johansson et al. 1989; Garrett 1978). Urine analysis revealed an elevated 9*R*-HHC-COOH concentration, supporting the positive cannabis immunoassay result on admission; it could be attributed to the well-known cross-reactivity with this carboxylic metabolite (Patton et al. 2024). The relative abundance of metabolites in blood and urine is consistent with previous studies reporting HHC-COOH as the major metabolite in plasma and HHC-OH-glucuronides in urine (Kronstrand et al. 2024; Lindbom et al. 2024). Due to the suspicion of meningoencephalitis, a cerebrospinal fluid sample was collected. This sample, obtained 14 h after exposure showed no detectable HHC (LOD 0.2 ng/mL). However, 9*R*-HHC-COOH was identified at a concentration of 9 ng/mL. The presence of this HHC metabolite may be explained by blood contamination resulting from the subarachnoid hemorrhage, as supported by its detection in plasma at 22 ng/mL.

In summary, using an untargeted analytical approach combined with molecular networking, HHC was identified as the most likely drug responsible for rhabdomyolysis in our patient, whose pattern of use was also characterized. The management of such intoxications generally relies solely on clinical presentation and patient history, as, in the absence of targeted toxicological testing, the involvement of a SC is typically established by exclusion (Kourouni et al. 2020; D’Errico et al. 1936; Paul et al. 2016).

### Clinical symptoms

Drug-induced rhabdomyolysis can be related to the direct action of the drug or mediated by various factors including prolonged muscle compression in a comatose patient, prolonged seizures, severe trauma, or metabolic disturbances (Huerta-Alardín et al. 2004; Chavez et al. 2016). Obesity has been also acknowledge as a risk factor (Chan et al. 2014). Seizures and recreational drugs including SCs are established triggers of rhabdomyolysis (Kourouni et al. 2020; Amanollahi et al. 2023; Waldman et al. 2021; Adedinsewo et al. 2016; AlKhateeb et al. 2025; Warren et al. 2002). However, to the best of our knowledge, this is the first case resulting from HHC intoxication.

Diagnosis of rhabdomyolysis is routinely confirmed by markedly elevated muscle injury biomarkers including CK (peak at 166,000 IU/L on D3 post-exposure in our patient), plasma myoglobin (less readily available), AST (usually exceeding ALT) and troponin Ic (mild elevation) (Huerta-Alardín et al. 2004; Stahl et al. 2020; Kodadek et al. 2022). AKI is a well-recognized complication of rhabdomyolysis, occurring in 5–25% of cases, and its severity correlated with plasma CK levels (Waldman et al. 2021; Ward 1553; Grossman et al. 1974). Decreased serum BUN/creatinine ratio, commonly observed in rhabdomyolysis-induced AKI, is attributed to the faster rise in creatinine compared to BUN in the presence of muscle injury (Walid 2008; Uchino et al. 2012). The patient’s renal function deteriorated rapidly with a BUN/creatinine ratio below 10:1 on admission reaching a nadir of 5:1 (normal range, 10:1–20:1). Interestingly, creatinine and CK concentrations were strongly correlated, highlighting the strong relationship between muscle damage and renal function. About 20% of patients with rhabdomyolysis-induced AKI may die (Huerta-Alardín et al. 2004), mainly from serum potassium disturbances. In our patient, no marked hyperkalemia was observed; serum potassium rose moderately, peaking at 5.4 mM, with no dysrhythmia. Subsequent to the diagnosis, the patient's plasma endometabolome was analyzed to assess the metabolic disruptions induced by the intoxication.

### Endometabolome analysis

Correlations between concentrations of 9*R*-HHC and endogenous metabolites suggest a possible role of amino acids in HHC poisoning. Consistent, alterations in phenylalanine and kynurenine pathways have been reported in the rat, a few hours after exposure to Δ^9^-THC (Maayah et al. 2022). Impaired phenylalanine metabolism was attributed to a decrease in phenylalanine hydroxylase activity. In our case, HHC significantly altered phenylalanine, phenylpyruvic acid and hippurate but not tyrosine concentrations (Grümer 1961). Citrate, a key intermediate of the Krebs cycle was also correlated with 9*R*-HHC. It plays a central role in cellular metabolism by exerting negative feedback on glycolysis through the inhibition of key glycolytic enzymes (Williams and O’Neill 2018). Besides in vitro study that has shown that phenylalanine and phenylpyruvic acid inhibit hepatic glycolysis (Suzuki et al. 2021), these correlations indicate an alteration of the glycolytic metabolism after HHC consumption. In vitro*,* alterations in glucose and fatty acid metabolism have previously been reported, highlighting a significant involvement of the CB1 receptor (Esposito et al. 2008; Eckardt et al. 2009). In addition, it should be emphasized that CB1 receptors are localized on mitochondrial membranes of striated muscles, and that their activation regulate Kreb’s cycle activity (Mendizabal-Zubiaga et al. 2016). Thus, by activating CB1 receptors and altering biochemical pathways and, through Kreb’s cycle and ATP metabolism, HHC may directly alter skeletal muscle tissues, in addition to seizures and subsequent tissue hypoxia and muscle immobilization (Schirmer et al. 2023; Garrett 1978; Patton et al. 2024; Esposito et al. 2008).

Contribution of hypermyoglobinemia to rhabdomyolysis-induced AKI has been well documented (Knochel 1993). In the present study, significant correlations between creatinine, tryptophan and kynurenic acid suggested that tryptophan catabolism is markedly upregulated during rhabdomyolysis-induced AKI, leading to increased production of kynurenic acid. Such mechanisms mediated by pro-inflammatory cytokines have been demonstrated in animal models of AKI (Wee et al. 2021): kynurenic acid, recognized for its anti-inflammatory, antioxidant and free radical-scavenging properties, accumulates during AKI as a result of reduced tubular clearance, thereby contributing to cellular protection. Thiamine and taurine, also correlated with creatinine in our study, have been described as nephroprotective agents in rhabdomyolysis-induced AKI (Al-Kharashi et al. 2023; Zamorskii et al. 2016). Similarly, pyridoxine and its metabolites, known to improve functional recovery after AKI (Skrypnyk et al. 2016), have been correlated with creatinine. The positive correlation of these metabolites with creatinine and CK in our study may correspond to protective mechanisms activated in response to rhabdomyolysis and resulting renal tissue injury.

Carnitine, a key amino acid-derived compound, plays a crucial role in mitochondrial fatty acid metabolism through β-oxidation. The inverse correlation with plasma creatinine likely reflects renal dysfunction, as carnitine is freely filtered by the glomerulus and about 90% is reabsorbed in the proximal tubules (Xiang et al. 2025). In conditions where β-oxidation is impaired or disrupted, carnitine utilization increases to facilitate the buffering and export of accumulating fatty acyl-CoA species, resulting in the accumulation of medium and long-chain acylcarnitines in the plasma (McCann et al. 2021). Thus, decrease in plasma carnitine level in rhabdomyolysis-induced AKI may be related to both β-oxidation disruption and renal impairment. Consequently, alterations in carnitine/acylcarnitine profile may serve as a surrogate marker of mitochondrial dysfunction and AKI (Dambrova et al. 2022). Consistently, a strong correlation between six dicarboxylic acids in plasma and both creatinine and CK levels was found. These diacids originate in ω-oxidation, occurring in endoplasmic reticulum, and which is upregulated when mitochondrial β-oxidation is impaired (Miura 2013; Begriche et al. 2011; Grünig et al. 2018). Elevated levels of these dicarboxylic acids reflect disturbances in lipid metabolism, suggesting a secondary metabolic compensation during rhabdomyolysis-induced AKI. Drug-induced alteration in β-oxidation or mitochondrial membrane integrity is known to affect fatty acid metabolism (Massart et al. 2013). To our knowledge, this is the first study to report evidence supporting the involvement of ω-oxidation in drug-related rhabdomyolysis-induced AKI.

### Limitations

This study has several technical limitations that should be raised. All measurements were collected longitudinally in a single patient, so successive values are not independent; Spearman correlations may therefore reflect temporal associations rather than independent statistical relationships, and p-values should be interpreted with caution. Samples were stored at −20 °C and underwent only a single freeze–thaw cycle prior to processing. While formal stability testing was not performed, some degree of degradation cannot be entirely excluded. However, because all samples were handled and processed together under identical conditions, any potential degradation would be systematic across the dataset, thereby minimizing its impact on the interpretation of relative temporal changes. Methadone-D3 was used as a single internal standard to correct for variability during pre-analytical extraction, although multiple standards could have been used to perform class-specific normalization. However, as neither absolute quantification nor comparison between the metabolites themselves is claimed, we considered that a single standard normalization is sufficient to study the longitudinal evolution of each metabolite.

## Conclusion

We reported a case of tonic–clonic seizure following HHC exposure complicated by severe rhabdomyolysis-induced AKI. The originality of our work includes a comprehensive analytical approach using untargeted metabolomic investigations combined with molecular networking that confirmed exposure to HHC as unique toxicant. Additionally, the exhaustive annotation of HHC metabolites allowed to characterize the patient’s consumption profile. Correlation analyses showed marked disturbances in various metabolisms related to HHC exposure, seizure activity and/or rhabdomyolysis-induced AKI. In particular, we identified associations between HHC exposure and phenylalanine pathway disruption and between impaired mitochondrial β-oxidation and rhabdomyolysis-induced AKI. Compensatory anti-inflammatory responses together with activation of secondary metabolic pathways including ω-oxidation, were reported in response to HHC exposure. This integrative analytical approach may be regarded as a complete workflow for characterizing drug intoxications and provides valuable insights into the clinical toxicodynamic of emerging psychoactive substances such as NPS and (S)SCs.

## Supplementary Information

Additional file 1: Instrument conditions.

Additional file 2: Chromatogram of 9*R* and 9*S*-HHC.

Additional file 3: List of phase I and phase II metabolites of HHC detected by UHPLC-ESI^+^-HRMS, arranged by increasing *m/z*.

Additional file 4: Spearman correlation with 9*R*-HHC, creatinine and CK concentrations.

## Data Availability

Datasets generated during and/or analyzed during the current study are available from the corresponding author on reasonable request.
