# Supplementary material for: Hexahydrocannabinol-induced rhabdomyolysis and acute kidney injury: a case report combining comprehensive toxicokinetic and metabolomic investigations
Source: J Cannabis Res. 2026 May 9;8:78. doi: 10.1186/s42238-026-00435-7 (PMC13326361; doi:10.1186/s42238-026-00435-7)
Supplement: Supplementary file 1 — Additional file 1: Instrument conditions. [file 42238_2026_435_MOESM1_ESM.docx]

**Additional File 1:** Instrument conditions.

| **E-liquids and vaping products analysis by GC-MS** | Chromatographic separation was performed on a CP-Sil 8 CB column (25 m x 0.25 cm, 0.25 µm, Agilent Technologies, Les Ulis, France). The injection is carried out in split mode with a ratio of 1:33 at 275 °C. The carrier gas was helium, in constant flow mode at a flow rate of 1.5 mL/min. A temperature gradient was applied during 35 min with a maximum at 280 °C obtained after 20 min and held for 15 min. The transfer line and the ion source temperature were set at 275 and 280 °C respectively. The scan time was 0.3 s and ranged between *m/z* 40 and 500. |
| --- | --- |
| **Quantitative targeted analysis** | Chromatographic separation of cannabinoids was achieved on a Acquity CSH C18® column (100 x 2.1 mm, 1.7 µm) (Waters Saint Quentin en Yvelines, France). Oven and autosampler were thermostated at 60 and 10 °C respectively. Mobile phase A and mobile phase B were composed of water (A) or MeOH (B) containing 0.1 % (*v/v*) of FA. Elution gradient was set as follows: 0–0.2 min 50 % B, 0.2–1.0 min from 50 % to 70 % B, 1.0–7.0 min from 70 % to 95 % B, 7.0–8.0 min 95 % B, 8.0–8.1 min from 95 % to 50 % B and 8.1–10.0 min 50 % B. The total run-time per sample was 10.0 min, equilibration included. Mobile phases were pumped at a flow rate of 0.4 mL/min.  MS analysis was performed using electrospray ionization in positive ion mode. Parameters of the source were set as follows: auxiliary gas heater temperature at 400°C, capillary temperature at 320°C, spray voltage at 3.5 kV, sheath gas flow rate 20 and auxiliary gas flow rate 12 arbitrary units. Data were acquired in single ion monitoring (SIM) scan mode, with an inclusion list of previously mentioned cannabinoids, and resolution was 35,000. Resolution of MS2 spectra was 17,000 with an isolation window of 2 *m/z*. |
| **Metabolomic analysis: xenometabolome** | Chromatographic condition were identical to those mentioned in the previous section - Quantitative targeted analysis. The electrospray ionization probe was operated in a polarity switching mode with a spray voltage at 3.5 kV for positive ion mode and 2.5 kV for negative ion mode. Data were acquired in full scan mode, with discovery data-dependant MS2 (ddMS2), *i.e* acquisition of three MS/MS spectra of the three most intense ions. Resolution was set at 35,000 for full scan mode, and at 17,000 for MS/MS scans with an isolation window of 2 *m/z*. The dynamic exclusion mode used was set at 3 s for each selected ion. |
| **Metabolomic analysis: endometabolome** | The column used was an Accucore® Phenyl Hexyl (100 x 2.1 mm, 2.6 µm) (Thermo Fisher Scientific) maintained at 40 °C. The flow rate of mobile phases was set at 0.5 mL/min. Mobile phases consisted of water (A) and MeOH/ACN (50/50, *v/v*, B) with 2 mM of formate ammonium and 0.1 % of FA. Gradient elution was set as follows: 0-1 min 1 % B, 1-10 min from 1 % to 99 % B, 10-11.5 min 99 % B, 11.5-11.6 min from 99 % to 1 % B, and 11.6-15.5 min 1 % B.  The electrospray ionization probe of the mass spectrometer operated in switching mode, and the parameters were set as follows: auxiliary gas heater temperature and capillary temperature at 320°C, spray voltage at 3.0 kV for positive ion mode and 2.5 kV for negative ion mode, sheath gas flow rate 35 and auxiliary gas flow rate 15 arbitrary units. Data were acquired in full scan, with discovery data-dependant MS2 (ddMS2) mode as mentioned for endometabolomic analysis with the exception that full scan ranged from *m/z* 100 and 1,000. |
