# Supplementary figures and images for: Hexahydrocannabinol-induced rhabdomyolysis and acute kidney injury: a case report combining comprehensive toxicokinetic and metabolomic investigations
Source: J Cannabis Res. 2026 May 9;8:78. doi: 10.1186/s42238-026-00435-7 (PMC13326361; doi:10.1186/s42238-026-00435-7)

**Additional File 2**: Chromatogram reconstituted at *m/z* 317.2481, showing 9*R* and 9*S*-HHC separation.


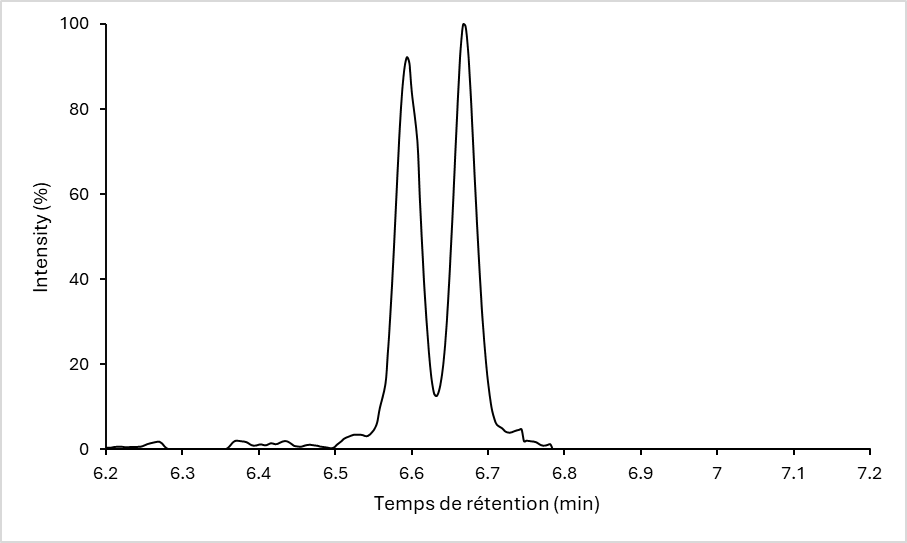


9*R*-HHC

9*S*-HHC

Supplement: Supplementary file 2 — Additional file 2: Chromatogram of 9R and 9S-HHC. [file 42238_2026_435_MOESM2_ESM.docx]
