# Supplementary material for: Hexahydrocannabinol-induced rhabdomyolysis and acute kidney injury: a case report combining comprehensive toxicokinetic and metabolomic investigations
Source: J Cannabis Res. 2026 May 9;8:78. doi: 10.1186/s42238-026-00435-7 (PMC13326361; doi:10.1186/s42238-026-00435-7)
Supplement: Supplementary file 3 — Additional file 3: List of phase I and phase II metabolites of HHC detected by UHPLC-ESI+-HRMS, arranged by increasing m/z. [file 42238_2026_435_MOESM3_ESM.docx]

**Additional File 3:** List of phase I and phase II metabolites of HHC detected by UHPLC-ESI^+^-HRMS, arranged by increasing *m/z.*

| ***m/z*** | **Proposed annotation (MSI level)** | **Proposed modification** | **Product ions of interest (*m/z*)** | **Retention time (min)** |
| --- | --- | --- | --- | --- |
| 291.1591 [M+H]^+^ | **Hexahydrocannabiorcol-COOH (3)** | Dealkylation (CH_2_)_4_ and carboxylation of the terminal carbon of the methyl chain | 207.0656 181.0498 167.0338 137.1324 137.0598 123.0441 107.0494 95.0861 | 3.88 |
| 319.1904 [M+H]^+^ | **Hexahydrocannabivarin-COOH (3)** | Dealkylation (CH_2_)_2_ and carboxylation of the terminal carbon of the propyl chain | 205.0872 191.0702 177.0545 165.0543 137.1324 123.0440 107.0491 95.0859 | 3.78 |
| 333.2423 [M+H]^+^  315.2319 [M-H_2_O+H]^+^ | **HHC-OH (3)** | Hydroxylation of the pentyl chain | 315.2310 205.1220 191.1066 137.1323 137.0596 123.0442 95.0859 | 4.55 |
| 333.2414 [M+H]^+^  315.2307 [M-H_2_O+H]^+^ | **HHC-OH (3)** | Hydroxylation of the pentyl chain | 315.2316 205.1226 191.1065 179.1076 137.1326 137.0597 123.0443 95.0860 | 4.73 |
| 333.2427 [M+H]^+^ | **HHC-OH (3)** | Hydroxylation of the cyclohexyl | 315.2319 207.1379 193.1223 181.1221 135.1167 123.0443 93.0705 | 4.99 |
| 333.2424 [M+H]^+^ | **HHC-OH (3)** | Hydroxylation of the cyclohexyl | 315.2325 207.1378 193.1222 181.1218 123.0441 135.1165 93.0704 | 5.12 |
| 335.1847 [M+H]^+^ | **Hexahydrocannabivarin-OH-COOH (3)** | Dealkylation (CH_2_)_2_ and carboxylation of the terminal carbon of the propyl chain + hydroxylation of the cyclohexyl | 317.1728 195.0651 177.0547 165.0541 135.1172 123.0445 93.0704 | 1.81 |
| 335.1847 [M+H]^+^ | **Hexahydrocannabivarin-OH-COOH (3)** | Dealkylation (CH_2_)_2_ and carboxylation of the terminal carbon of the propyl chain + hydroxylation of the cyclohexyl | 317.1745 195.0651 177.0545 165.0541 135.1166 123.0441 93.0704 | 2.10 |
| 335.1852 [M+H]^+^ | **Hexahydrocannabivarin-OH-COOH (3)** | Dealkylation (CH_2_)_2_ and carboxylation of the terminal carbon of the propyl chain + hydroxylation of the cyclohexyl | 317.1749 195.0651 177.0545 165.0549 135.1171 123.0442 93.0704 | 2.37 |
| 347.2219 [M+H]^+^ | **9*R*-HHC-COOH (1)** | Carboxylation of C11 | 329.2106 311.2002 207.1013 193.1223 181.1223 123.0444 95.0860 93.0704 | 4.90 |
| 347.2220 [M+H]^+^ | **HHC-COOH (3)** | Carboxylation of C11 | 329.2096 311.2011 207.1020 193.1222 123.0443 95.0861 93.0704 | 5.15 |
| 349.2375 [M+H]^+^ | **HHC-(OH)_2_ (3)** | Hydroxylation of the pentyl chain and the cyclohexyl | 331.2271 313.2154 205.1226 191.1065 179.1066 135.1170 123.0442 93.0704 | 2.47 |
| 349.2373 [M+H]^+^ | **HHC-(OH)_2_ (3)** | Hydroxylation of the pentyl chain and the cyclohexyl | 331.2267 313.2157 205.1220 191.1066 179.1073 135.1171 123.0439 93.0703 | 2.68 |
| 349.2373 [M+H]^+^ | **HHC-(OH)_2_ (3)** | Hydroxylation of the pentyl chain and the cyclohexyl | 331.2265 313.2158 205.1221 191.1066 179.1067 123.0443 135.1167 93.0703 | 2.93 |
| 349.2372 [M+H]^+^ | **HHC-(OH)_2_ (3)** | Hydroxylation of the pentyl chain and the cyclohexyl | 331.2253 205.1228 191.1065 135.1170 123.0444 93.0703 | 3.09 |
| 363.2166 [M+H]^+^ | **HHC-OH-COOH (3)** | Carboxylation of C11 + hydroxylation of the pentyl chain | 345.2056 327.1953 205.0853 191.1065 179.1065 123.0443 135.1178 93.0704 | 2.86 |
| 363.2165 [M+H]^+^  345.2062 [M-H_2_O+H]^+^ | **HHC-OH-COOH (3)** | Carboxylation of C11 + hydroxylation of the pentyl chain | 345.2056 205.0860 191.1065 179.1058 123.0443 93.0706 | 3.03 |
| 363.2166 [M+H]^+^ | **HHC-OH-COOH (3)** | Carboxylation of C11 + hydroxylation of the cyclohexyl | 345.2048 327.1964 207.1380 193.1227 181.1222 123.0445 95.0862 93.0706 | 3.40 |
| 419.2432 | *Unidentified* |  | 329.2115 311.2016 301.2161 259.1698 193.1223 181.1221 207.1017 123.0443 121.1015 93.0704 | 5.35 |
| 435.2379 | *Unidentified* |  | 417.2268 361.1627 327.1952 243.1371 191.1065 205.0857 121.1014 | 3.12 |
| 435.2381 | *Unidentified* |  | 417.2272 243.1371 191.1065 205.0856 121.1015 | 3.18 |
| 435.2380 | *Unidentified* |  | 417.2259 361.1668 327.1947 243.1379 191.1063 205.0852 121.1013 | 3.34 |
| 493.2800 [M+H]^+^  317.2476 [M-Gluc+H]^+^ | **HHC-Gluc (3)** |  | 317.2473 207.1380 193.1225 137.1323 123.0441 95.0860 | 5.36 |
| 493.2798 [M+H]^+^ | **HHC-Gluc (3)** |  | 317.2477 207.1382 193.1223 95.0861 | 5.61 |
| 509.2749 [M+H]^+^  333.2422 [M-Gluc+H]^+^ | **HHC-OH-Gluc (3)** | Hydroxylation of the cyclohexyl | 333.2420 315.2313 207.1380 193.1223 181.1229 123.0442 135.1170 93.0704 | 3.07 |
| 509.2750 [M+H]^+^ | **HHC-OH-Gluc (3)** | Hydroxylation of the pentyl | 333.2426 315.2313 191.1065 137.1328 123.0440 95.0859 | 3.34 |
| 509.2750 [M+H]^+^  333.2423 [M-Gluc+H]^+^ | **HHC-OH-Gluc (3)** | Hydroxylation of the cyclohexyl | 333.2416 207.1378 193.1221 123.0448 135.1168 93.0702 | 4.22 |
| 509.2749 [M+H]^+^ | **HHC-OH-Gluc (3)** | Hydroxylation of the cyclohexyl | 333.2418 315.2316 207.1381 193.1222 181.1226 123.0442 135.1171 93.0703 | 4.39 |
| 509.2752 [M+H]^+^ | **HHC-OH-Gluc (3)** | Hydroxylation of the cyclohexyl | 333.2421 315.2313 207.1379 193.1222 181.1228 135.1170 123.0442 93.0705 | 4.46 |
| 509.2750 [M+H]^+^ | **HHC-OH-Gluc (3)** | Hydroxylation of the cyclohexyl | 333.2422 315.2320 207.1376 193.1222 135.1170 123.0442 93.0705 | 4.57 |
| 511.2179 [M+H]^+^ | **Hexahydrocannabivarin-OH-COOH-Gluc (3)** | Dealkylation (CH_2_)_2_ and carboxylation of the terminal carbon of the propyl chain + hydroxylation of the cyclohexyl | 335.1848 209.0801 195.0650 177.0545 149.0598 135.1168 123.0441 93.0705 | 2.05 |
| 523.2542 [M+H]^+^ | **HHC-COOH-Gluc (3)** | Carboxylation of C11 | 505.2430 347.2209 329.2097 311.1994 207.1018 193.1223 181.1222 123.0442 93.0702 | 4.05 |
| 523.2543 [M+H]^+^  347.2216 [M-Gluc+H]^+^ | **HHC-COOH-Gluc (3)** | Carboxylation of C11 | 505.2430 347.2213 329.2103 311.2003 207.1017 193.1223 181.1223 123.0442 93.0705 | 4.16 |
| 525.2703 [M+H]^+^ | **HHC-(OH)_2_-Gluc (3)** | Hydroxylation of the pentyl chain and the cyclohexyl | 349.2368 331.2242 313.2166 191.1065 135.1170 123.0441 93.0705 | 1.57 |
| 525.2703 [M+H]^+^ | **HHC-(OH)_2_-Gluc (3)** | Hydroxylation of the pentyl chain and the cyclohexyl | 349.2351 331.2260 313.2150 191.1070 135.1170 123.0443 93.0704 | 1.96 |
| 525.2695 [M+H]^+^ | **HHC-(OH)_2_-Gluc (3)** | Hydroxylation of the pentyl chain and the cyclohexyl | 349.2372 331.2266 313.2163 205.1220 191.1067 179.1067 135.1170 123.0442 93.0704 | 2.63 |
| 525.2708 [M+H]^+^ | **HHC-(OH)_2_-Gluc (3)** | Hydroxylation of the pentyl chain and the cyclohexyl | 349.2377 331.2263 313.2166 205.1221 191.1065 179.1068 135.1168 123.0441 93.0705 | 2.8 |
| 525.2700 [M+H]^+^ | **HHC-(OH)_2_-Gluc (3)** | Dihydroxylation of the cyclohexyl | 349.2373 331.2258 313.2153 207.1382 193.1224 181.1224 123.0440 | 2.91 |
| 539.2489 [M+H]^+^ | **HHC-OH-COOH-Gluc (3)** | Carboxylation on C11 + hydroxylation on the pentyl chain | 363.2158 345.2059 327.1958 223.0962 205.0860 191.1065 177.0910 135.1169 93.0705 | 2.48 |

*MSI : Metabolomics Standards Initiative*
