# Supplementary material for: Hexahydrocannabinol-induced rhabdomyolysis and acute kidney injury: a case report combining comprehensive toxicokinetic and metabolomic investigations
Source: J Cannabis Res. 2026 May 9;8:78. doi: 10.1186/s42238-026-00435-7 (PMC13326361; doi:10.1186/s42238-026-00435-7)
Supplement: Supplementary file 4 — Additional file 4: Spearman correlation with 9R-HHC, creatinine and CK concentrations. [file 42238_2026_435_MOESM4_ESM.docx]

**Additional File 4:**

**Additional Table 1:** Spearman correlation with 9*R*-HHC concentrations.

| **HMDB** | **Name** | **Spearman r** | **P value summary** | **Adjusted p-value** | **Number of XY Pairs** |
| --- | --- | --- | --- | --- | --- |
| HMDB0013676 | 2,6-dihydroxybenzoic acid | 0.93 | **** | <0.0001 | 17 |
| HMDB0036083 | CARVEOL | 0.89 | *** | 0.0005 | 17 |
| HMDB0000077 | Dehydroepiandrosterone DHEA | 0.85 | ** | 0.0017 | 17 |
| HMDB0033624 | 2-Hydroxy-3-(4-hydroxyphenyl)propanoic acid | 0.85 | ** | 0.0015 | 17 |
| HMDB0011134 | 5-HETE | 0.84 | ** | 0.0014 | 17 |
| HMDB0000159 | *L*-phenylalanine | 0.83 | ** | 0.0017 | 17 |
| HMDB0000205 | Acide phenylpyruvique | 0.83 | ** | 0.0019 | 17 |
| HMDB0000671 | Indolelactic acid | 0.79 | ** | 0.0057 | 17 |
| HMDB0002825 | Theobromine | 0.79 | ** | 0.0050 | 17 |
| HMDB0000289 | Urate | 0.76 | ** | 0.0090 | 17 |
| HMDB0011103 | 1,7-dimethyluric acid | 0.76 | ** | 0.0085 | 17 |
| HMDB0000112 | 4-aminobutyric acid | 0.74 | * | 0.0139 | 17 |
| HMDB0000715 | kynurenic acid | 0.73 | * | 0.0147 | 17 |
| HMDB0061112 | CMPF | 0.72 | * | 0.0160 | 17 |
| HMDB0001518 | Alfa_CEHC | 0.72 | * | 0.0156 | 17 |
| HMDB0000094 | Citrate | 0.72 | * | 0.0148 | 17 |
| HMDB0011718 | 4-hydroxybenzaldehyde | 0.68 | * | 0.0313 | 17 |
| HMDB0001046 | Cotinine | 0.66 | * | 0.0368 | 17 |
| HMDB0000714 | Hippurate | 0.64 | * | 0.0498 | 17 |
| HMDB0000792 | Sebacic acid | 0.63 | ns | 0.0513 | 17 |
| HMDB0000201 | Acetylcarnitine | 0.63 | ns | 0.0529 | 17 |
| HMDB0000182 | *L*-Lysine | 0.59 | ns | 0.0822 | 17 |
| HMDB0062180 | *N*-lactoylisoleucine | 0.58 | ns | 0.0950 | 17 |
| HMDB0014693 | Methoxsalen | 0.57 | ns | 0.0994 | 17 |
| HMDB0000132 | guanine | 0.56 | ns | 0.1023 | 17 |
| HMDB0004160 | Urobilin | 0.55 | ns | 0.1131 | 17 |
| HMDB0000157 | Hypoxanthine | 0.54 | ns | 0.1198 | 17 |
| HMDB0013248 | MEHP | 0.53 | ns | 0.1279 | 17 |
| HMDB0062175 | *N*-lactoyl-phenylalanine | 0.52 | ns | 0.1323 | 17 |
| HMDB0000555 | 3-Methyladipic acid | 0.52 | ns | 0.1348 | 17 |
| HMDB0000070 | Pipecolic acid | 0.51 | ns | 0.1389 | 17 |
| HMDB0001868 | 5-Methoxysalicylic Acid | 0.50 | ns | 0.1522 | 17 |
| HMDB0000933 | Traumatic acid | 0.50 | ns | 0.1555 | 17 |
| HMDB0032985 | 5-(2-Hydroxyethyl)-4-methylthiazole | 0.49 | ns | 0.1580 | 17 |
| HMDB0000623 | Dodecanedioic acid | 0.49 | ns | 0.1584 | 17 |
| HMDB0341381 | Pyroglutamyl-Isoleucine | 0.48 | ns | 0.1674 | 17 |
| HMDB0000893 | Suberic acid | 0.48 | ns | 0.1745 | 17 |
| HMDB0003681 | 4-Acetamidobutyric acid | 0.48 | ns | 0.1729 | 17 |
| HMDB0000122 | glucose | 0.47 | ns | 0.1757 | 17 |
| HMDB0247961 | Aconitic acid | 0.47 | ns | 0.1738 | 17 |
| HMDB0006294 | 16-hydroxypalmitic acid | 0.47 | ns | 0.1739 | 17 |
| HMDB0002092 | Itaconic acid | 0.46 | ns | 0.1962 | 17 |
| HMDB0341110 | Camphanic acid | 0.45 | ns | 0.1994 | 17 |
| HMDB0000292 | Xanthine | 0.45 | ns | 0.2026 | 17 |
| HMDB0034276 | cyclo-(L-Leu-L-Pro) | 0.44 | ns | 0.2213 | 17 |
| HMDB0005066 | Tetradecanoylcarnitine | 0.43 | ns | 0.2295 | 17 |
| HMDB0001860 | Paraxanthine | 0.43 | ns | 0.2300 | 17 |
| HMDB0004824 | N2_N2-Dimethylguanosine | 0.42 | ns | 0.2378 | 17 |
| HMDB0000784 | Azelaic acid | 0.41 | ns | 0.2636 | 17 |
| HMDB0000017 | 4-pyridoxate | 0.40 | ns | 0.2647 | 17 |
| HMDB0009003 | PE(18:0/20:4) | 0.40 | ns | 0.2789 | 17 |
| HMDB0000517 | *L*-arginine | 0.39 | ns | 0.2972 | 17 |
| HMDB0002250 | LAUROYLCARNITINE | 0.38 | ns | 0.3007 | 17 |
| HMDB0000036 | Taurocholic acid | 0.37 | ns | 0.3217 | 17 |
| HMDB0000235 | Thiamine | 0.37 | ns | 0.3317 | 17 |
| HMDB0000872 | Tetradecanedioic acid | 0.36 | ns | 0.3496 | 17 |
| HMDB0002183 | cis-4,7,10,13,16,19-Docosahexaenoic acid (DHA) | 0.35 | ns | 0.3749 | 17 |
| HMDB0000172 | Isoleucine | 0.34 | ns | 0.3904 | 17 |
| HMDB0000512 | *N*-acetyl-phenylalanine | 0.33 | ns | 0.3907 | 17 |
| HMDB0008946 | PE(16:0/22:6) | 0.33 | ns | 0.3889 | 17 |
| HMDB0000158 | *L*-Tyrosine | 0.33 | ns | 0.3925 | 17 |
| HMDB0001043 | Arachidonic acid | 0.33 | ns | 0.3887 | 17 |
| HMDB0284419 | (2-aminoethoxy)[(2*R*)-2-[(4*Z*,7*Z*,10*Z*,13*Z*,16*Z*,19*Z*)-docosa-4,7,10,13,16,19-hexaenoyloxy]-3-(octadecanoyloxy)propoxy]phosphinic acid | 0.32 | ns | 0.3914 | 17 |
| HMDB0012611 | 18-HEPE | 0.32 | ns | 0.3923 | 17 |
| HMDB0000239 | Pyridoxine | 0.28 | ns | 0.4644 | 17 |
| HMDB0243890 | LPC(18:0) | 0.27 | ns | 0.4912 | 17 |
| [HMDB0009059](https://hmdb.ca/metabolites/HMDB0009059) | PE(18:1/18:1) | 0.27 | ns | 0.4931 | 17 |
| HMDB0240259 | Stercobilin | 0.26 | ns | 0.5028 | 17 |
| HMDB0062588 | O-(2-tetradecenoyl)carnitine | 0.26 | ns | 0.5048 | 17 |
| HMDB0006344 | Phenylacetyl-glutamine | 0.25 | ns | 0.5134 | 17 |
| HMDB0000687 | Leucine | 0.25 | ns | 0.5155 | 17 |
| HMDB0000195 | Inosine | 0.23 | ns | 0.5379 | 17 |
| HMDB0000791 | Octanoylcarnitine | 0.23 | ns | 0.5357 | 17 |
| HMDB0004679 | 8-HETE | 0.22 | ns | 0.5672 | 17 |
| HMDB0251169 | Dibenzylamine | 0.21 | ns | 0.6013 | 17 |
| HMDB0000631 | glycodeoxycholic acid | 0.20 | ns | 0.6100 | 17 |
| [HMDB0007965](https://hmdb.ca/metabolites/HMDB0007965) | PC(16:0/14:0) | 0.19 | ns | 0.6344 | 17 |
| HMDB0012958 | gamma Tocotrienol | 0.17 | ns | 0.6837 | 17 |
| HMDB0011394 | PE(P-18:0/22:6) | 0.17 | ns | 0.6897 | 17 |
| HMDB0001847 | Caffeine | 0.15 | ns | 0.7182 | 17 |
| HMDB0012458 | 7alpha-hydroxy-3-oxo-4-cholestenoic acid | 0.14 | ns | 0.7529 | 17 |
| HMDB0000711 | 2-HYDROXYOCTANOIC ACID | 0.14 | ns | 0.7543 | 17 |
| HMDB0007971 | PC(16:0/18:1) | 0.12 | ns | 0.8136 | 17 |
| HMDB0000068 | Epinephrine | 0.12 | ns | 0.8140 | 17 |
| HMDB0000254 | Succinic acid | 0.11 | ns | 0.8169 | 17 |
| HMDB0000842 | 2-quinoline carboxylic acid | 0.11 | ns | 0.8258 | 17 |
| HMDB0029377 | Piperine | 0.10 | ns | 0.8554 | 17 |
| [HMDB0005048](https://hmdb.ca/metabolites/HMDB0005048) | Conjugated linoleic Acid (10E,12Z) | 0.10 | ns | 0.8517 | 17 |
| HMDB0009027 | PE(18:1/18:2) | 0.09 | ns | 0.8532 | 17 |
| HMDB0061115 | lenticin | 0.09 | ns | 0.8605 | 17 |
| HMDB0000874 | Tauroursodeoxycholic acid | 0.09 | ns | 0.8607 | 17 |
| HMDB0000696 | *L-*methionine | 0.09 | ns | 0.8552 | 17 |
| HMDB0000651 | Decanoylcarnitine | 0.07 | ns | 0.8986 | 17 |
| HMDB0000883 | Valine | 0.07 | ns | 0.9041 | 17 |
| HMDB0001008 | Biliverdin | 0.06 | ns | 0.9150 | 17 |
| [HMDB0012108](https://hmdb.ca/metabolites/HMDB0012108) | PC(17:0) | 0.05 | ns | 0.9421 | 17 |
| [HMDB0010379](https://hmdb.ca/metabolites/HMDB0010379) | LysoPC(14:0/0:0) | 0.05 | ns | 0.9391 | 17 |
| HMDB0005783 | 6-Gingerol | 0.04 | ns | 0.9474 | 17 |
| HMDB0000688 | Isovalerylcarnitine | 0.02 | ns | 0.9838 | 17 |
| HMDB0244507 | 13-Docosenamide | 0.02 | ns | 0.9940 | 17 |
| HMDB0007973 | PC(16:0/18:2) | 0.02 | ns | 0.9895 | 17 |
| HMDB0000251 | Taurine | 0.02 | ns | 0.9895 | 17 |
| HMDB0000138 | Glycocholic acid | 0.02 | ns | 0.9765 | 17 |
| HMDB0000684 | *L-*kynurenine | 0.01 | ns | 0.9948 | 17 |
| HMDB0000222 | Palmitoylcarnitine | 0.00 | ns | 0.9945 | 17 |
| HMDB0008097 | PC(18:1/14:0) | 0.00 | ns | 0.9933 | 17 |
| HMDB0008937 | PE(16:0/20:4) | -0.01 | ns | 0.9966 | 17 |
| HMDB0000097 | Choline | -0.03 | ns | 0.9668 | 17 |
| HMDB0002013 | Butyrylcarnitine | -0.04 | ns | 0.9415 | 17 |
| [HMDB0010381](https://hmdb.ca/metabolites/HMDB0010381) | LPC(15:0) | -0.04 | ns | 0.9414 | 17 |
| [HMDB0010382](https://hmdb.ca/metabolites/HMDB0010382) | LPC(16:0) | -0.06 | ns | 0.9057 | 17 |
| HMDB0010169 | SM(d18:1/16:0) | -0.09 | ns | 0.8633 | 17 |
| HMDB0009684 | PE(22:6/18:0) | -0.14 | ns | 0.7543 | 17 |
| HMDB0013302 | Phe-Phe | -0.14 | ns | 0.7571 | 17 |
| HMDB0029737 | 3-Formylindole | -0.16 | ns | 0.7005 | 17 |
| HMDB0000925 | Trimethylamine *N*-oxide | -0.17 | ns | 0.6899 | 17 |
| HMDB0006750 | C16 Lactosyl Ceramide (d18:1/16:0) | -0.17 | ns | 0.6918 | 17 |
| [HMDB0011503](https://hmdb.ca/metabolites/HMDB0011503) | LPE(16:0) | -0.20 | ns | 0.6073 | 17 |
| HMDB0000888 | Undecanedioic acid | -0.21 | ns | 0.5935 | 17 |
| [HMDB0002815](https://hmdb.ca/metabolites/HMDB0002815) | LPC(18:1) | -0.24 | ns | 0.5313 | 17 |
| HMDB0010387 | LPC (18:3) | -0.24 | ns | 0.5253 | 17 |
| HMDB0031404 | Cyclohexylamine | -0.25 | ns | 0.5150 | 17 |
| HMDB0005065 | Oleoyl-*L*-Carnitine | -0.26 | ns | 0.5053 | 17 |
| HMDB0001085 | 5,12-DiHETE | -0.26 | ns | 0.5070 | 17 |
| HMDB0033244 | Mdibutylphthalate | -0.26 | ns | 0.5053 | 17 |
| HMDB0000824 | Propionylcarnitine | -0.26 | ns | 0.5011 | 17 |
| HMDB0000067 | Cholesterol | -0.28 | ns | 0.4668 | 17 |
| HMDB0240607 | glycohyocholic acid | -0.28 | ns | 0.4696 | 17 |
| HMDB0001895 | Salicylic acid | -0.29 | ns | 0.4459 | 17 |
| HMDB0244272 | 10-hydroxydecanoic acid | -0.30 | ns | 0.4280 | 17 |
| HMDB0240493 | cyclo(*L*-Val-*L*-Pro) | -0.31 | ns | 0.4032 | 17 |
| HMDB0002327 | Tridecanedioic acid | -0.31 | ns | 0.4049 | 17 |
| HMDB0013609 | Tryptophan | -0.33 | ns | 0.3891 | 17 |
| HMDB0000637 | Glycochenodeoxycholate | -0.34 | ns | 0.3861 | 17 |
| HMDB0000062 | Carnitine | -0.34 | ns | 0.3897 | 17 |
| HMDB0249243 | DEHP | -0.34 | ns | 0.3915 | 17 |
| [HMDB0245120](https://hmdb.ca/metabolites/HMDB0245120) | 2-Ethylhexyl diphenyl phosphate | -0.42 | ns | 0.2376 | 17 |
| HMDB0248866 | (*R*)-4-((3*R*,5*R*,8*R*,9*S*,10*S*,12*S*,13*R*,14*S*,17*R*)-3,12-dihydroxy-10,13-dimethylhexadecahydro-1H-cyclopenta[a]phenanthren-17-yl)pent-2-enoic acid | -0.52 | ns | 0.1372 | 17 |
| HMDB0010386 | LPC (18:2) | -0.53 | ns | 0.1307 | 17 |
| HMDB0240635 | SM(d18:2/24:0) | -0.54 | ns | 0.1218 | 17 |
| HMDB0000305 | Retinol | -0.57 | ns | 0.0980 | 17 |
| HMDB0000518 | chenodeoxycholic acid | -0.58 | ns | 0.0934 | 17 |
| HMDB0000626 | Deoxycholic acid | -0.59 | ns | 0.0846 | 17 |
| [HMDB0011506](https://hmdb.ca/metabolites/HMDB0011506) | PE(18:1) | -0.60 | ns | 0.0756 | 17 |
| HMDB0000619 | cholic acid | -0.65 | * | 0.0438 | 17 |

**Additional Table 2:** Spearman correlation with creatinine concentrations.

| **HMDB** | **Name** | **Spearman r** | **P value summary** | **Adjusted p-value** | **Number of XY Pairs** |
| --- | --- | --- | --- | --- | --- |
| HMDB0032985 | 5-(2-Hydroxyethyl)-4-methylthiazole | 0.95 | <0.0001 | **** | 17 |
| HMDB0000235 | Thiamine | 0.94 | <0.0001 | **** | 17 |
| HMDB0006344 | Phenylacetyl-glutamine | 0.93 | <0.0001 | **** | 17 |
| HMDB0240259 | Stercobilin | 0.93 | <0.0001 | **** | 17 |
| HMDB0000017 | 4-pyridoxate | 0.91 | <0.0001 | **** | 17 |
| HMDB0003681 | 4-Acetamidobutyric acid | 0.91 | <0.0001 | **** | 17 |
| HMDB0000068 | Epinephrine | 0.91 | <0.0001 | **** | 17 |
| HMDB0001868 | 5-Methoxysalicylic Acid | 0.90 | <0.0001 | **** | 17 |
| HMDB0004824 | N2_N2-Dimethylguanosine | 0.86 | 0.0003 | *** | 17 |
| HMDB0341110 | Camphanic acid | 0.86 | 0.0003 | *** | 17 |
| HMDB0000512 | *N*-acetyl-phenylalanine | 0.85 | 0.0004 | *** | 17 |
| HMDB0000239 | Pyridoxine | 0.84 | 0.0005 | *** | 17 |
| HMDB0000555 | 3-Methyladipic acid | 0.84 | 0.0005 | *** | 17 |
| HMDB0000792 | Sebacic acid | 0.84 | 0.0005 | *** | 17 |
| HMDB0014693 | Methoxsalen | 0.83 | 0.0006 | *** | 17 |
| HMDB0000842 | 2-quinoline carboxylic acid | 0.83 | 0.0006 | *** | 17 |
| HMDB0000872 | Tetradecanedioic acid | 0.82 | 0.0008 | *** | 17 |
| HMDB0000784 | Azelaic acid | 0.81 | 0.0011 | ** | 17 |
| HMDB0000132 | guanine | 0.80 | 0.0017 | ** | 17 |
| HMDB0000715 | kynurenic acid | 0.78 | 0.0026 | ** | 17 |
| HMDB0000893 | Suberic acid | 0.77 | 0.0030 | ** | 17 |
| HMDB0000251 | Taurine | 0.75 | 0.0050 | ** | 17 |
| HMDB0004160 | Urobilin | 0.74 | 0.0057 | ** | 17 |
| HMDB0001518 | Alfa_CEHC | 0.73 | 0.0063 | ** | 17 |
| HMDB0011103 | 1,7-dimethyluric acid | 0.73 | 0.0066 | ** | 17 |
| HMDB0000631 | glycodeoxycholic acid | 0.72 | 0.0075 | ** | 17 |
| HMDB0000714 | Hippurate | 0.69 | 0.0114 | * | 17 |
| HMDB0000933 | Traumatic acid | 0.67 | 0.0157 | * | 17 |
| HMDB0000623 | Dodecanedioic acid | 0.66 | 0.0167 | * | 17 |
| HMDB0034276 | cyclo-(*L-*Leu-*L-*Pro) | 0.66 | 0.0167 | * | 17 |
| HMDB0011718 | 4-hydroxybenzaldehyde | 0.65 | 0.0213 | * | 17 |
| HMDB0002250 | LAUROYLCARNITINE | 0.61 | 0.0366 | * | 17 |
| HMDB0033624 | 2-Hydroxy-3-(4-hydroxyphenyl)propanoic acid | 0.60 | 0.0371 | * | 17 |
| HMDB0062588 | O-(2-tetradecenoyl)carnitine | 0.60 | 0.0371 | * | 17 |
| HMDB0005066 | Tetradecanoylcarnitine | 0.59 | 0.0420 | * | 17 |
| HMDB0000094 | Citrate | 0.58 | 0.0464 | * | 17 |
| HMDB0000925 | Trimethylamine *N*-oxide | 0.53 | 0.0826 | ns | 17 |
| HMDB0036083 | CARVEOL | 0.51 | 0.1007 | ns | 17 |
| HMDB0284419 | (2-aminoethoxy)[(2*R*)-2-[(4*Z*,7*Z*,10*Z*,13*Z*,16*Z*,19*Z*)-docosa-4,7,10,13,16,19-hexaenoyloxy]-3-(octadecanoyloxy)propoxy]phosphinic acid | 0.50 | 0.1069 | ns | 17 |
| HMDB0000688 | Isovalerylcarnitine | 0.49 | 0.1234 | ns | 17 |
| HMDB0062180 | *N*-lactoylisoleucine | 0.48 | 0.1262 | ns | 17 |
| HMDB0012611 | 18-HEPE | 0.46 | 0.1557 | ns | 17 |
| HMDB0009684 | PE(22:6/18:0) | 0.46 | 0.1569 | ns | 17 |
| HMDB0004679 | 8-HETE | 0.45 | 0.1640 | ns | 17 |
| HMDB0001043 | Arachidonic acid | 0.42 | 0.2020 | ns | 17 |
| HMDB0008937 | PE(16:0/20:4) | 0.41 | 0.2282 | ns | 17 |
| HMDB0000077 | Dehydroepiandrosterone DHEA | 0.40 | 0.2396 | ns | 17 |
| HMDB0006294 | 16-hydroxypalmitic acid | 0.38 | 0.2643 | ns | 17 |
| HMDB0002092 | Itaconic acid | 0.38 | 0.2684 | ns | 17 |
| HMDB0000205 | Acide phenylpyruvique | 0.38 | 0.2775 | ns | 17 |
| HMDB0000671 | Indolelactic acid | 0.38 | 0.2775 | ns | 17 |
| HMDB0247961 | Aconitic acid | 0.37 | 0.2731 | ns | 17 |
| HMDB0011134 | 5-HETE | 0.37 | 0.2718 | ns | 17 |
| HMDB0000289 | Urate | 0.37 | 0.2754 | ns | 17 |
| HMDB0000791 | Octanoylcarnitine | 0.36 | 0.2791 | ns | 17 |
| HMDB0008946 | PE(16:0/22:6) | 0.35 | 0.2973 | ns | 17 |
| HMDB0000651 | Decanoylcarnitine | 0.34 | 0.3088 | ns | 17 |
| HMDB0000122 | glucose | 0.34 | 0.3108 | ns | 17 |
| HMDB0002183 | cis-4,7,10,13,16,19-Docosahexaenoic acid (DHA) | 0.34 | 0.3185 | ns | 17 |
| HMDB0012958 | gamma Tocotrienol | 0.33 | 0.3239 | ns | 17 |
| HMDB0341381 | Pyroglutamyl-Isoleucine | 0.31 | 0.3552 | ns | 17 |
| HMDB0001008 | Biliverdin | 0.28 | 0.3990 | ns | 17 |
| HMDB0000112 | 4-aminobutyric acid | 0.28 | 0.3962 | ns | 17 |
| HMDB0000157 | Hypoxanthine | 0.27 | 0.4296 | ns | 17 |
| HMDB0062175 | *N*-lactoyl-phenylalanine | 0.27 | 0.4391 | ns | 17 |
| HMDB0013676 | 2,6-dihydroxybenzoic acid | 0.25 | 0.4750 | ns | 17 |
| HMDB0000159 | *L*-phenylalanine | 0.25 | 0.4843 | ns | 17 |
| HMDB0005783 | 6-Gingerol | 0.23 | 0.5299 | ns | 17 |
| HMDB0011503 | LPE(16:0) | 0.23 | 0.5322 | ns | 17 |
| HMDB0031404 | Cyclohexylamine | 0.21 | 0.5807 | ns | 17 |
| HMDB0000201 | Acetylcarnitine | 0.20 | 0.5973 | ns | 17 |
| HMDB0000182 | *L*-Lysine | 0.20 | 0.5929 | ns | 17 |
| HMDB0010169 | SM(d18:1/16:0) | 0.19 | 0.6195 | ns | 17 |
| HMDB0002825 | Theobromine | 0.18 | 0.6427 | ns | 17 |
| HMDB0011394 | PE(P-18:0/22:6) | 0.18 | 0.6396 | ns | 17 |
| HMDB0000222 | Palmitoylcarnitine | 0.17 | 0.6594 | ns | 17 |
| HMDB0000874 | Tauroursodeoxycholic acid | 0.15 | 0.7096 | ns | 17 |
| HMDB0013248 | MEHP | 0.14 | 0.7344 | ns | 17 |
| HMDB0000254 | Succinic acid | 0.10 | 0.8424 | ns | 17 |
| HMDB0061112 | CMPF | 0.09 | 0.8621 | ns | 17 |
| HMDB0000687 | Leucine | 0.06 | 0.9161 | ns | 17 |
| HMDB0005048 | Conjugated linoleic Acid (10*E*,12*Z*) | 0.06 | 0.9172 | ns | 17 |
| HMDB0000696 | *L*-methionine | 0.04 | 0.9535 | ns | 17 |
| HMDB0009027 | PE(18:1/18:2) | 0.04 | 0.9535 | ns | 17 |
| HMDB0000195 | Inosine | 0.04 | 0.9314 | ns | 17 |
| HMDB0001860 | Paraxanthine | 0.04 | 0.9365 | ns | 17 |
| HMDB0005065 | Oleoyl-*L*-Carnitine | 0.02 | 0.9752 | ns | 17 |
| HMDB0000517 | *L*-arginine | 0.02 | 0.9745 | ns | 17 |
| HMDB0007971 | PC(16:0/18:1) | 0.02 | 0.9701 | ns | 17 |
| HMDB0002013 | Butyrylcarnitine | 0.02 | 0.9709 | ns | 17 |
| HMDB0001895 | Salicylic acid | 0.00 | 0.9887 | ns | 17 |
| HMDB0001085 | 5,12-DiHETE | -0.01 | 0.9879 | ns | 17 |
| HMDB0001847 | Caffeine | -0.01 | 0.9774 | ns | 17 |
| HMDB0000292 | Xanthine | -0.02 | 0.9761 | ns | 17 |
| HMDB0248866 | (*R*)-4-((3*R*,5*R*,8*R*,9*S*,10*S*,12*S*,13*R*,14*S*,17*R*)-3,12-dihydroxy-10,13-dimethylhexadecahydro-1H-cyclopenta[a]phenanthren-17-yl)pent-2-enoic acid | -0.04 | 0.9368 | ns | 17 |
| HMDB0000036 | Taurocholic acid | -0.04 | 0.9409 | ns | 17 |
| HMDB0000067 | Cholesterol | -0.05 | 0.9338 | ns | 17 |
| HMDB0000172 | Isoleucine | -0.06 | 0.9185 | ns | 17 |
| HMDB0010382 | LPC(16:0) | -0.07 | 0.9161 | ns | 17 |
| HMDB0007965 | PC(16:0/14:0) | -0.07 | 0.9157 | ns | 17 |
| HMDB0001046 | Cotinine | -0.09 | 0.8607 | ns | 17 |
| HMDB0011506 | PE(18:1) | -0.10 | 0.8409 | ns | 17 |
| HMDB0006750 | C16 Lactosyl Ceramide (d18:1/16:0) | -0.10 | 0.8364 | ns | 17 |
| HMDB0009059 | PE(18:1/18:1) | -0.11 | 0.8293 | ns | 17 |
| HMDB0245120 | 2-Ethylhexyl diphenyl phosphate | -0.12 | 0.7957 | ns | 17 |
| HMDB0000888 | Undecanedioic acid | -0.13 | 0.7566 | ns | 17 |
| HMDB0000637 | Glycochenodeoxycholate | -0.14 | 0.7324 | ns | 17 |
| HMDB0249243 | DEHP | -0.15 | 0.7075 | ns | 17 |
| HMDB0009003 | PE(18:0/20:4) | -0.16 | 0.7019 | ns | 17 |
| HMDB0000070 | Pipecolic acid | -0.20 | 0.5940 | ns | 17 |
| HMDB0008097 | PC(18:1/14:0) | -0.30 | 0.3688 | ns | 17 |
| HMDB0000138 | Glycocholic acid | -0.31 | 0.3526 | ns | 17 |
| HMDB0000158 | *L*-Tyrosine | -0.31 | 0.3570 | ns | 17 |
| HMDB0000305 | Retinol | -0.31 | 0.3599 | ns | 17 |
| HMDB0002327 | Tridecanedioic acid | -0.31 | 0.3526 | ns | 17 |
| HMDB0000684 | *L*-kynurenine | -0.32 | 0.3476 | ns | 17 |
| HMDB0251169 | Dibenzylamine | -0.32 | 0.3395 | ns | 17 |
| HMDB0007973 | PC(16:0/18:2) | -0.33 | 0.3304 | ns | 17 |
| HMDB0000619 | cholic acid | -0.33 | 0.3206 | ns | 17 |
| HMDB0029737 | 3-Formylindole | -0.34 | 0.3072 | ns | 17 |
| HMDB0244272 | 10-hydroxydecanoic acid | -0.35 | 0.3086 | ns | 17 |
| HMDB0243890 | LPC(18:0) | -0.37 | 0.2772 | ns | 17 |
| HMDB0240635 | SM(d18:2/24:0) | -0.37 | 0.2736 | ns | 17 |
| HMDB0061115 | lenticin | -0.39 | 0.2513 | ns | 17 |
| HMDB0000824 | Propionylcarnitine | -0.41 | 0.2172 | ns | 17 |
| HMDB0033244 | Mdibutylphthalate | -0.43 | 0.1998 | ns | 17 |
| HMDB0244507 | 13-Docosenamide | -0.47 | 0.1396 | ns | 17 |
| HMDB0012458 | 7alpha-hydroxy-3-oxo-4-cholestenoic acid | -0.48 | 0.1256 | ns | 17 |
| HMDB0012108 | PC(17:0) | -0.52 | 0.0882 | ns | 17 |
| HMDB0002815 | LPC(18:1) | -0.53 | 0.0859 | ns | 17 |
| HMDB0010386 | LPC (18:2) | -0.60 | 0.0387 | * | 17 |
| HMDB0240607 | glycohyocholic acid | -0.60 | 0.0360 | * | 17 |
| HMDB0000097 | Choline | -0.61 | 0.0337 | * | 17 |
| HMDB0000711 | 2-HYDROXYOCTANOIC ACID | -0.63 | 0.0276 | * | 17 |
| HMDB0013302 | Phe-Phe | -0.64 | 0.0240 | * | 17 |
| HMDB0000626 | Deoxycholic acid | -0.68 | 0.0136 | * | 17 |
| HMDB0000518 | chenodeoxycholic acid | -0.68 | 0.0133 | * | 17 |
| HMDB0240493 | cyclo(*L*-Val-*L*-Pro) | -0.70 | 0.0105 | * | 17 |
| HMDB0029377 | Piperine | -0.71 | 0.0091 | ** | 17 |
| HMDB0000883 | Valine | -0.71 | 0.0094 | ** | 17 |
| HMDB0013609 | Tryptophan | -0.71 | 0.0087 | ** | 17 |
| HMDB0010379 | LysoPC(14:0/0:0) | -0.71 | 0.0088 | ** | 17 |
| HMDB0010381 | LPC(15:0) | -0.73 | 0.0064 | ** | 17 |
| HMDB0010387 | LPC (18:3) | -0.84 | 0.0005 | *** | 17 |
| HMDB0000062 | Carnitine | -0.90 | <0.0001 | **** | 17 |

**Additional Table 3:** Spearman correlation with CK concentrations.

| **HMDB** | **Name** | **Spearman r** | **P value summary** | **Adjust p-value** | **Number of XY Pairs** |
| --- | --- | --- | --- | --- | --- |
| HMDB0000017 | 4-pyridoxate | 0.99 | *** | 0.0008 | 10 |
| HMDB0000235 | Thiamine | 0.99 | *** | 0.0008 | 10 |
| HMDB0032985 | 5-(2-Hydroxyethyl)-4-methylthiazole | 0.98 | ** | 0.0010 | 10 |
| HMDB0003681 | 4-Acetamidobutyric acid | 0.95 | ** | 0.0032 | 10 |
| HMDB0006344 | Phenylacetyl-glutamine | 0.95 | ** | 0.0032 | 10 |
| HMDB0240259 | Stercobilin | 0.95 | ** | 0.0032 | 10 |
| HMDB0033624 | 2-Hydroxy-3-(4-hydroxyphenyl)propanoic acid | 0.94 | ** | 0.0033 | 10 |
| HMDB0000555 | 3-Methyladipic acid | 0.94 | ** | 0.0033 | 10 |
| HMDB0001868 | 5-Methoxysalicylic Acid | 0.94 | ** | 0.0033 | 10 |
| HMDB0341110 | Camphanic acid | 0.94 | ** | 0.0033 | 10 |
| HMDB0014693 | Methoxsalen | 0.92 | ** | 0.0042 | 10 |
| HMDB0000792 | Sebacic acid | 0.92 | ** | 0.0056 | 10 |
| HMDB0000132 | guanine | 0.89 | * | 0.0105 | 10 |
| HMDB0004824 | N2_N2-Dimethylguanosine | 0.89 | * | 0.0112 | 10 |
| HMDB0000715 | kynurenic acid | 0.88 | * | 0.0137 | 10 |
| HMDB0011103 | 1,7-dimethyluric acid | 0.88 | * | 0.0139 | 10 |
| HMDB0000842 | 2-quinoline carboxylic acid | 0.87 | * | 0.0167 | 10 |
| HMDB0000784 | Azelaic acid | 0.87 | * | 0.0167 | 10 |
| HMDB0004160 | Urobilin | 0.87 | * | 0.0167 | 10 |
| HMDB0000068 | Epinephrine | 0.86 | * | 0.0152 | 10 |
| HMDB0000631 | glycodeoxycholic acid | 0.84 | * | 0.0235 | 10 |
| HMDB0000893 | Suberic acid | 0.84 | * | 0.0235 | 10 |
| HMDB0000872 | Tetradecanedioic acid | 0.83 | * | 0.0275 | 10 |
| HMDB0000251 | Taurine | 0.82 | * | 0.0327 | 10 |
| HMDB0000239 | Pyridoxine | 0.81 | * | 0.0367 | 10 |
| HMDB0000512 | *N*-acetyl-phenylalanine | 0.81 | * | 0.0363 | 10 |
| HMDB0062180 | *N*-lactoylisoleucine | 0.80 | * | 0.0376 | 10 |
| HMDB0001518 | Alfa_CEHC | 0.80 | * | 0.0344 | 10 |
| HMDB0034276 | cyclo-(*L*-Leu-*L*-Pro) | 0.77 | ns | 0.0513 | 10 |
| HMDB0036083 | CARVEOL | 0.76 | ns | 0.0623 | 10 |
| HMDB0000623 | Dodecanedioic acid | 0.75 | ns | 0.0706 | 10 |
| HMDB0000205 | Acide phenylpyruvique | 0.71 | ns | 0.0976 | 10 |
| HMDB0000933 | Traumatic acid | 0.71 | ns | 0.0976 | 10 |
| HMDB0062175 | *N*-lactoyl-phenylalanine | 0.70 | ns | 0.0913 | 10 |
| HMDB0000289 | Urate | 0.70 | ns | 0.0992 | 10 |
| HMDB0000714 | Hippurate | 0.60 | ns | 0.2144 | 10 |
| HMDB0000077 | Dehydroepiandrosterone DHEA | 0.59 | ns | 0.2179 | 10 |
| HMDB0012958 | gamma Tocotrienol | 0.59 | ns | 0.2179 | 10 |
| HMDB0002250 | LAUROYLCARNITINE | 0.58 | ns | 0.2261 | 10 |
| HMDB0011134 | 5-HETE | 0.55 | ns | 0.2552 | 10 |
| HMDB0005066 | Tetradecanoylcarnitine | 0.55 | ns | 0.2552 | 10 |
| HMDB0002092 | Itaconic acid | 0.54 | ns | 0.2589 | 10 |
| HMDB0004679 | 8-HETE | 0.54 | ns | 0.2639 | 10 |
| HMDB0000157 | Hypoxanthine | 0.54 | ns | 0.2639 | 10 |
| HMDB0000094 | Citrate | 0.53 | ns | 0.2765 | 10 |
| HMDB0011718 | 4-hydroxybenzaldehyde | 0.52 | ns | 0.2757 | 10 |
| HMDB0000671 | Indolelactic acid | 0.52 | ns | 0.2907 | 10 |
| HMDB0062588 | O-(2-tetradecenoyl)carnitine | 0.52 | ns | 0.2907 | 10 |
| HMDB0005783 | 6-Gingerol | 0.50 | ns | 0.3048 | 10 |
| HMDB0013248 | MEHP | 0.46 | ns | 0.3728 | 10 |
| HMDB0000122 | glucose | 0.45 | ns | 0.3773 | 10 |
| HMDB0000688 | Isovalerylcarnitine | 0.45 | ns | 0.3773 | 10 |
| HMDB0001847 | Caffeine | 0.44 | ns | 0.3821 | 10 |
| HMDB0000292 | Xanthine | 0.44 | ns | 0.3876 | 10 |
| HMDB0247961 | Aconitic acid | 0.43 | ns | 0.3982 | 10 |
| HMDB0000925 | Trimethylamine *N*-oxide | 0.43 | ns | 0.3982 | 10 |
| HMDB0012611 | 18-HEPE | 0.42 | ns | 0.4090 | 10 |
| HMDB0001008 | Biliverdin | 0.42 | ns | 0.4090 | 10 |
| HMDB0000182 | *L*-Lysine | 0.42 | ns | 0.4090 | 10 |
| HMDB0000517 | *L*-arginine | 0.39 | ns | 0.4482 | 10 |
| HMDB0000791 | Octanoylcarnitine | 0.38 | ns | 0.4574 | 10 |
| HMDB0341381 | Pyroglutamyl-Isoleucine | 0.38 | ns | 0.4574 | 10 |
| HMDB0000201 | Acetylcarnitine | 0.37 | ns | 0.4692 | 10 |
| HMDB0001043 | Arachidonic acid | 0.37 | ns | 0.4692 | 10 |
| HMDB0000651 | Decanoylcarnitine | 0.37 | ns | 0.4692 | 10 |
| HMDB0001860 | Paraxanthine | 0.36 | ns | 0.4708 | 10 |
| HMDB0000254 | Succinic acid | 0.35 | ns | 0.4873 | 10 |
| HMDB0013676 | 2,6-dihydroxybenzoic acid | 0.30 | ns | 0.5712 | 10 |
| HMDB0002183 | cis-4,7,10,13,16,19-Docosahexaenoic acid (DHA) | 0.28 | ns | 0.5882 | 10 |
| HMDB0000159 | *L*-phenylalanine | 0.28 | ns | 0.5882 | 10 |
| HMDB0002825 | Theobromine | 0.28 | ns | 0.5882 | 10 |
| HMDB0008946 | PE(16:0/22:6) | 0.27 | ns | 0.6004 | 10 |
| HMDB0000874 | Tauroursodeoxycholic acid | 0.25 | ns | 0.6468 | 10 |
| [HMDB0005048](https://hmdb.ca/metabolites/HMDB0005048) | Conjugated linoleic Acid (10*E*,12*Z*) | 0.24 | ns | 0.6634 | 10 |
| HMDB0284419 | (2-aminoethoxy)[(2*R*)-2-[(4*Z*,7*Z*,10*Z*,13*Z*,16*Z*,19*Z*)-docosa-4,7,10,13,16,19-hexaenoyloxy]-3-(octadecanoyloxy)propoxy]phosphinic acid | 0.22 | ns | 0.6873 | 10 |
| HMDB0000687 | Leucine | 0.21 | ns | 0.6991 | 10 |
| [HMDB0011503](https://hmdb.ca/metabolites/HMDB0011503) | LPE(16:0) | 0.20 | ns | 0.7222 | 10 |
| HMDB0061112 | CMPF | 0.16 | ns | 0.7859 | 10 |
| [HMDB0010382](https://hmdb.ca/metabolites/HMDB0010382) | LPC(16:0) | 0.15 | ns | 0.7936 | 10 |
| HMDB0000195 | Inosine | 0.14 | ns | 0.8194 | 10 |
| HMDB0006294 | 16-hydroxypalmitic acid | 0.13 | ns | 0.8139 | 10 |
| HMDB0000172 | Isoleucine | 0.13 | ns | 0.8296 | 10 |
| HMDB0008937 | PE(16:0/20:4) | 0.13 | ns | 0.8296 | 10 |
| HMDB0011394 | PE(P-18:0/22:6) | 0.13 | ns | 0.8296 | 10 |
| HMDB0002013 | Butyrylcarnitine | 0.10 | ns | 0.8618 | 10 |
| HMDB0001085 | 5,12-DiHETE | 0.09 | ns | 0.8774 | 10 |
| HMDB0009684 | PE(22:6/18:0) | 0.08 | ns | 0.8999 | 10 |
| [HMDB0245120](https://hmdb.ca/metabolites/HMDB0245120) | 2-Ethylhexyl diphenyl phosphate | 0.06 | ns | 0.9094 | 10 |
| HMDB0000112 | 4-aminobutyric acid | 0.04 | ns | 0.9521 | 10 |
| HMDB0000696 | *L*-methionine | -0.01 | ns | 1.0000 | 10 |
| HMDB0001046 | Cotinine | -0.02 | ns | 0.9797 | 10 |
| HMDB0000684 | *L*-kynurenine | -0.02 | ns | 0.9661 | 10 |
| HMDB0000070 | Pipecolic acid | -0.03 | ns | 0.9658 | 10 |
| [HMDB0007965](https://hmdb.ca/metabolites/HMDB0007965) | PC(16:0/14:0) | -0.05 | ns | 0.9232 | 10 |
| HMDB0010169 | SM(d18:1/16:0) | -0.07 | ns | 0.9087 | 10 |
| HMDB0000888 | Undecanedioic acid | -0.07 | ns | 0.9067 | 10 |
| HMDB0248866 | (*R*)-4-((3*R*,5*R*,8*R*,9*S*,10*S*,12*S*,13*R*,14*S*,17*R*)-3,12-dihydroxy-10,13-dimethylhexadecahydro-1H-cyclopenta[a]phenanthren-17-yl)pent-2-enoic acid | -0.07 | ns | 0.9089 | 10 |
| HMDB0000637 | Glycochenodeoxycholate | -0.10 | ns | 0.8618 | 10 |
| HMDB0000036 | Taurocholic acid | -0.10 | ns | 0.8593 | 10 |
| HMDB0033244 | Mdibutylphthalate | -0.14 | ns | 0.8157 | 10 |
| [HMDB0009059](https://hmdb.ca/metabolites/HMDB0009059) | PE(18:1/18:1) | -0.15 | ns | 0.7967 | 10 |
| HMDB0031404 | Cyclohexylamine | -0.16 | ns | 0.7859 | 10 |
| HMDB0249243 | DEHP | -0.18 | ns | 0.7395 | 10 |
| HMDB0000222 | Palmitoylcarnitine | -0.19 | ns | 0.7451 | 10 |
| HMDB0001895 | Salicylic acid | -0.19 | ns | 0.7451 | 10 |
| HMDB0000067 | Cholesterol | -0.22 | ns | 0.6873 | 10 |
| HMDB0007971 | PC(16:0/18:1) | -0.22 | ns | 0.6873 | 10 |
| HMDB0000138 | Glycocholic acid | -0.24 | ns | 0.6462 | 10 |
| [HMDB0011506](https://hmdb.ca/metabolites/HMDB0011506) | PE(18:1) | -0.27 | ns | 0.6004 | 10 |
| HMDB0012458 | 7alpha-hydroxy-3-oxo-4-cholestenoic acid | -0.30 | ns | 0.5712 | 10 |
| HMDB0251169 | Dibenzylamine | -0.32 | ns | 0.5097 | 10 |
| HMDB0000097 | Choline | -0.33 | ns | 0.5092 | 10 |
| HMDB0009003 | PE(18:0/20:4) | -0.33 | ns | 0.5092 | 10 |
| HMDB0009027 | PE(18:1/18:2) | -0.33 | ns | 0.5092 | 10 |
| HMDB0029377 | Piperine | -0.35 | ns | 0.4828 | 10 |
| HMDB0000824 | Propionylcarnitine | -0.36 | ns | 0.4759 | 10 |
| HMDB0000305 | Retinol | -0.36 | ns | 0.4759 | 10 |
| HMDB0006750 | C16 Lactosyl Ceramide (d18:1/16:0) | -0.38 | ns | 0.4574 | 10 |
| HMDB0005065 | Oleoyl-*L*-Carnitine | -0.41 | ns | 0.4153 | 10 |
| HMDB0243890 | LPC(18:0) | -0.42 | ns | 0.4090 | 10 |
| HMDB0000158 | *L*-Tyrosine | -0.42 | ns | 0.4048 | 10 |
| HMDB0007973 | PC(16:0/18:2) | -0.44 | ns | 0.3876 | 10 |
| HMDB0013302 | Phe-Phe | -0.44 | ns | 0.3876 | 10 |
| HMDB0029737 | 3-Formylindole | -0.46 | ns | 0.3661 | 10 |
| HMDB0008097 | PC(18:1/14:0) | -0.49 | ns | 0.3186 | 10 |
| HMDB0240607 | glycohyocholic acid | -0.49 | ns | 0.3229 | 10 |
| HMDB0000711 | 2-HYDROXYOCTANOIC ACID | -0.56 | ns | 0.2424 | 10 |
| HMDB0240635 | SM(d18:2/24:0) | -0.56 | ns | 0.2424 | 10 |
| HMDB0002327 | Tridecanedioic acid | -0.59 | ns | 0.2179 | 10 |
| HMDB0000619 | cholic acid | -0.60 | ns | 0.2144 | 10 |
| HMDB0010386 | LPC (18:2) | -0.60 | ns | 0.2144 | 10 |
| [HMDB0012108](https://hmdb.ca/metabolites/HMDB0012108) | PC(17:0) | -0.60 | ns | 0.2144 | 10 |
| HMDB0244272 | 10-hydroxydecanoic acid | -0.61 | ns | 0.2008 | 10 |
| [HMDB0010381](https://hmdb.ca/metabolites/HMDB0010381) | LPC(15:0) | -0.66 | ns | 0.1349 | 10 |
| HMDB0244507 | 13-Docosenamide | -0.68 | ns | 0.1155 | 10 |
| HMDB0013609 | Tryptophan | -0.68 | ns | 0.1100 | 10 |
| HMDB0000518 | chenodeoxycholic acid | -0.71 | ns | 0.0976 | 10 |
| HMDB0000626 | Deoxycholic acid | -0.71 | ns | 0.0976 | 10 |
| [HMDB0010379](https://hmdb.ca/metabolites/HMDB0010379) | LysoPC(14:0/0:0) | -0.72 | ns | 0.0874 | 10 |
| HMDB0000883 | Valine | -0.73 | ns | 0.0777 | 10 |
| HMDB0240493 | cyclo(*L-*Val-*L*-Pro) | -0.75 | ns | 0.0706 | 10 |
| [HMDB0002815](https://hmdb.ca/metabolites/HMDB0002815) | LPC(18:1) | -0.77 | ns | 0.0539 | 10 |
| HMDB0061115 | lenticin | -0.81 | * | 0.0363 | 10 |
| HMDB0010387 | LPC (18:3) | -0.81 | * | 0.0363 | 10 |
| HMDB0000062 | Carnitine | -0.95 | ** | 0.0032 | 10 |
